# Single Amino Acid Exchange in ACTIN2 Confers Increased Tolerance to Oxidative Stress in Arabidopsis *der1–3* Mutant

**DOI:** 10.3390/ijms22041879

**Published:** 2021-02-13

**Authors:** Lenka Kuběnová, Tomáš Takáč, Jozef Šamaj, Miroslav Ovečka

**Affiliations:** Department of Cell Biology, Centre of the Region Haná for Biotechnological and Agricultural Research, Faculty of Science, Palacký University Olomouc, Šlechtitelů 27, 783 71 Olomouc, Czech Republic; lenka.kubenova@upol.cz (L.K.); tomas.takac@upol.cz (T.T.); jozef.samaj@upol.cz (J.Š.)

**Keywords:** ACTIN2, actin cytoskeleton, antioxidant capacity, Arabidopsis, *der1–3* mutant, lipid peroxidation, oxidative stress, root hairs, single amino acid exchange

## Abstract

Single-point mutation in the *ACTIN2* gene of the *der1–3* mutant revealed that ACTIN2 is an essential actin isovariant required for root hair tip growth, and leads to shorter, thinner and more randomly oriented actin filaments in comparison to the wild-type C24 genotype. The actin cytoskeleton has been linked to plant defense against oxidative stress, but it is not clear how altered structural organization and dynamics of actin filaments may help plants to cope with oxidative stress. In this study, we characterized root growth, plant biomass, actin organization and antioxidant activity of the *der1–3* mutant under oxidative stress induced by paraquat and H_2_O_2_. Under these conditions, plant growth was better in the *der1–3* mutant, while the actin cytoskeleton in the *der1–3* carrying *pro35S::GFP:FABD2* construct showed a lower bundling rate and higher dynamicity. Biochemical analyses documented a lower degree of lipid peroxidation, and an elevated capacity to decompose superoxide and hydrogen peroxide. These results support the view that the *der1–3* mutant is more resistant to oxidative stress. We propose that alterations in the actin cytoskeleton, increased sensitivity of ACTIN to reducing agent dithiothreitol (DTT), along with the increased capacity to decompose reactive oxygen species encourage the enhanced tolerance of this mutant against oxidative stress.

## 1. Introduction

Plants are continuously exposed to fluctuating environmental conditions, including adverse biotic and abiotic stressors. Oxidative stress, alone or in combination with other stress factors, may disrupt the cellular homeostasis in plants. Oxidative stress significantly increases symplastic and apoplastic amounts of reactive oxygen species (ROS) such as superoxide (O_2_^•−^), hydrogen peroxide (H_2_O_2_), hydroxyl radical (OH^•^) and singlet oxygen (^1^O_2_). Although ROS also serve as signaling molecules, playing important roles in the regulation of numerous plant developmental processes [1,2,3], they are generated as toxic by-products of the aerobic metabolism under stress conditions [4,5,6,7,8]. In general, the production of ROS with metabolic or stress-related origin, is controlled by components of redox signaling pathways. These maintain cellular ROS homeostasis, since both low and high ROS levels are undesirable for plant cells. Thus, an equilibrated threshold of ROS is maintained and controlled by the activity of antioxidant enzymes from the family of superoxide dismutases (SODs), catalases, peroxidases, gluthatione peroxidases, iron uptake/storage regulating proteins, and a network of thio- and glutaredoxins [8,9,10].

A common approach in inducing oxidative stress in plants experimentally is based on the external application of paraquat (PQ, 1,1′-dimethyl-4,4′-bipyridinium chloride) and hydrogen peroxide (H_2_O_2_). PQ, or methyl viologen, is a widely used herbicide, which passes rapidly to the cells [11,12]. PQ is effective particularly in photosynthetically-active plant tissues [13]. Primary place of its activity is chloroplast, where PQ takes away electrons, probably from photosystem I and ferredoxin. This process leads to the formation of stable reduced cationic radical, reacting rapidly with molecular oxygen to form O_2_^•−^ [14,15]. Superoxide anion interferes with antioxidant defense mechanisms, leading to the damage of cells due to numerous chain reactions [16]. Treatment of plants with PQ also affects gene expression. Alterations have been documented in the expression of numerous genes encoding different protein kinases such as receptor-like kinases (RLKs), mitogen-activated protein kinases (MAPKs) or calcium-dependent protein kinases (CDPKs), antioxidant enzymes such as ascorbate peroxidase, CuZn SOD (CSD), FeSOD (FSD), some transcription factors that contain typical DNA binding motifs such as MYB and MYC, or in genes securing cell structural integrity [17]. All these changes likewise lead to the significant oxidation of proteins or nucleic acids [18]. The typical phenotypical reaction of affected plants is wilting and chlorosis. Prolonged PQ exposure causes the browning of damaged tissues and severe chlorosis, leading to the leaves falling off [12].

H_2_O_2_ is generated in chloroplasts, mitochondria and peroxisomes as an inevitable by-product of aerobic metabolism, or it is produced under stress conditions (both biotic and abiotic); [19]. It is a relatively long-living molecule (up to 1 ms) with the ability to pass membranes either by diffusion of actively via aquaporins [20,21,22,23]. The biological activity of H_2_O_2_ is mediated by its ability to oxidize free SH groups [22]. Excessive exogenous H_2_O_2_ application induces rapid cell death and necrosis of plant cells, even without passing through the apoptotic stage [19,24,25]. The external application of H_2_O_2_ can stimulate some morphogenetic events in plants, such as adventitious root initiation in flax hypocotyls [26]. H_2_O_2_, particularly at higher concentrations, affects the expression pattern of genes involved in diverse plant defense responses. It can be exemplified by changing expression patterns of genes encoding biosynthetic enzymes of phenylpropanoids, lignin and salicylic acid [27,28], as well as enzymes protecting against oxidative stress, such as glutathione S-transferase (GST) [29] and anthranilate synthase (ASA1), which is required for the biosynthesis of the phytoalexin camalexin [30]. The excessive formation of H_2_O_2_ also causes oxidative impairments of photosynthetic apparatus [22,31,32].

Actin filaments are essential cytoskeletal components, playing important roles in cellular (e.g., cytoplasmic streaming, organelle movement, vesicular trafficking) and developmental processes (e.g., establishment and maintenance of cell polarity and shape, cell division plane determination, tip growth). A typical feature of the actin cytoskeleton is its ability to perform dynamic structural reorganizations [33,34]. The actin cytoskeleton is also involved in signaling events triggered by diverse external stimuli. Among others, actin cytoskeleton remodeling is part of abiotic stress response mechanisms in plants [35]. Interestingly, dysfunctional ACTIN2 isoform or destabilization of actin microfilaments using cytochalasin D alters the localization of Respiratory burst oxidase homolog protein C (RbohC) during root hair development in Arabidopsis [36]. The connection between the intracellular distribution pattern of NDC1 (NAD(P)H dehydrogenases type II), and its ability to reduce mitochondrial ROS production and ACTIN2 was also revealed [37]. These data suggest a supporting role of actin filaments in the mediation of both short- and long-term plant responses to oxidative stress conditions. The dependence between changing dynamics of the actin cytoskeleton and elevated ROS level was described in Arabidopsis root tip cells under salt stress. Treatment with NADPH oxidase inhibitor diphenyleneiodonium prevented a salt stress-induced ROS increase, but treatment with actin inhibitors latrunculin B or jasplakinolide caused an enhanced ROS accumulation in salt stress-treated root cells [38]. Actin microfilaments play an important role in vesicular trafficking, which is linking ROS signaling with auxin transport [39]. In the proposed model, oxidative stress caused by H_2_O_2_ affects the dynamics of the actin cytoskeleton, which subsequently interferes with the ADP-ribosylation factor guaninenucleotide exchange factor (ARF-GEF)-dependent trafficking of PIN2 from the plasma membrane to early endosomes [39]. However, the complete mechanism regulating the structural and dynamic properties of the actin cytoskeleton under oxidative stress in plants is not fully understood yet.

The actin cytoskeleton is essential for the tip growth of root hairs. Indispensable functions of actin in growing root hairs were documented by pharmacological [40] and genetic [41,42] means. Evolution of land plants brought a diversification into reproductive and vegetative classes of actin, later ones represented by ACTIN2, ACTIN7 and ACTIN8 [43,44]. Genetic approaches using chemically-induced single-point mutation [42] or insertional knockout mutation [41] revealed that *ACTIN2* is essential for proper root hair tip growth. Expression level of the *ACTIN2* gene is not affected by the single point mutations in the *DER1* (*DEFORMED ROOT HAIRS 1*) locus and wild-type levels of *ACTIN2* expression has been documented in the *der1* (*deformed root hairs1*) mutants [42]. Nevertheless, the palette of received mutants (*der1–1*, *der1–2*, *der1–3*) showed different degrees of the mutant root hair phenotype [42]. It provides an opportunity to characterize the involvement of ACTIN2, altered by single-point mutations in *der1* mutants [42], in different aspects of their development [45]. In this study, we describe growth and developmental parameters of the *der1–3* mutant, bearing a strong *ACTIN2* mutation phenotype [42], under oxidative stress caused by PQ and H_2_O_2_. In comparison to plants of the wild-type (C24 ecotype), post-germination root growth, biomass production, antioxidant activity and the prevention of lipid peroxidation were more effective in the *der1–3* mutant. Considering the lower bundling rate and higher dynamicity of the actin cytoskeleton, together with the increased capacity of ACTIN for redox modifications caused by reducing agent dithiothreitol (DTT), we conclude that *der1–3* mutant plants are more resistant to mild and severe oxidative stress induced by PQ or H_2_O_2_ in the culture medium.

## 2. Results

### 2.1. Impact of the der1–3 Mutation and Its Topology on Protein Tertiary Structure

**Figure 1:**
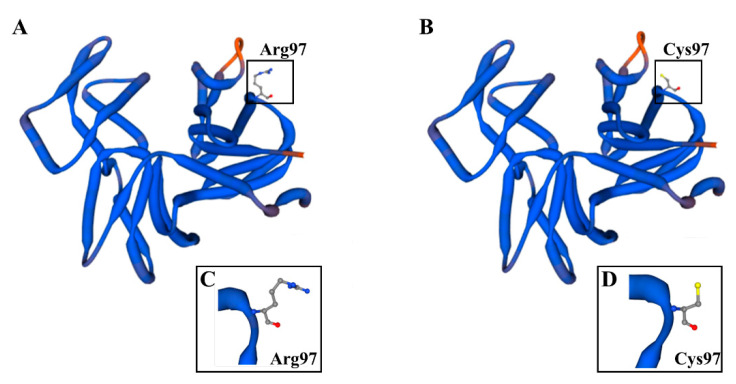
Model of the natural ACTIN2 protein structure and its mutated version in *der1–3* mutant. (**A**,**B**) SWISS model of the tertiary protein structure of ACTIN2 based on the wild-type gene sequence (**A**) or on the gene sequence altered by single-point mutation in the *der1–3* mutant (**B**). Topological location of arginine in the position 97 of the natural ACTIN2 (**A**) and substituted cysteine in the position 97 of the mutated ACTIN2 (**B**) of the *der1–3* mutant are showed in boxes. (**C**,**D**) Detailed structure of spatial arrangements of Arg97 (**C**) and Cys97 (**D**) from the boxed area in (**A**,**B**), respectively. 3D rotational models of protein structures are presented in Videos S1 and S2. Models of protein structures were produced in the application SWISS-MODEL (Biozentrum, University of Basel, Switzerland).

*Arabidopsis thaliana* mutant *der1–3* has been produced in the C24 ecotype background by an ethylmethanesulfonic acid-induced mutagenesis in the *DER1* locus, leading to a single-point mutation in the *ACTIN2* gene [42]. In general, *der1* mutants were selected according to disturbed root hair development phenotypes [42,46], and subsequent complementation analysis rescuing the normal root hair phenotype through expression of the *ACTIN2* cDNA under the control of *ACTIN2* or root hair-specific *CHIMERIC LEUCINE-RICH REPEAT/EXTENSIN1* (*LRX1)* promoters, confirming mutations in the *ACTIN2* gene [42]. Single-point mutation in the genomic DNA sequence of the gene was determined at the position 1114 (changing cytosine to thymine), leading to an altered protein sequence exchanging Arg97 to Cys97 in the *der1–3* mutant [42]. We translated a nucleotide sequence, both in a natural and mutated variant, to a primary protein sequence and we prepared a model of a tertiary protein structure. We found that this position, both in the natural (Figure 1A) and mutated (Figure 1B) ACTIN2 protein is placed in a loop located at the protein periphery (Videos S1 and S2). Importantly, based on the 3D model, the mutation does not alter the overall tertiary structure of the protein (Figure 1A,B), while this single amino acid exchange is topologically exposed to the protein surface (Figure 1C,D). This analysis indicates that single-point mutation exchanging Arg to Cys at the position 97 in the ACTIN2 protein of the *der1–3* mutant might rather influence its biochemical properties.

### 2.2. Influence of Oxidative Stress on Post-Germination Root Growth

**Figure 2:**
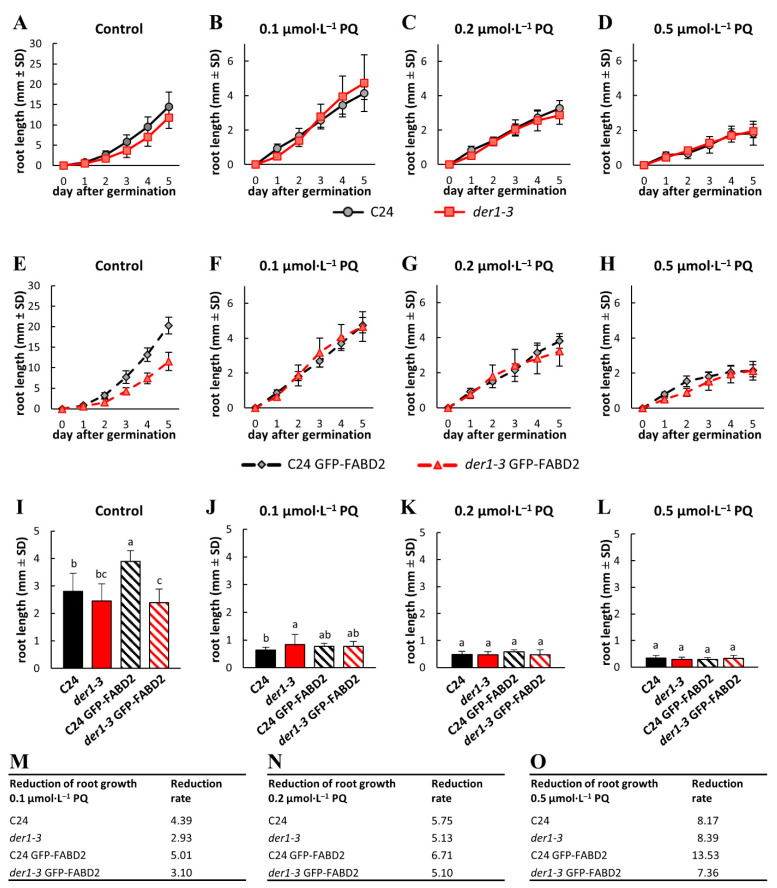
Root growth rate in plants of the control C24, *der1–3* mutant and transgenic C24 and *der1–3* lines expressing *pro35S::GFP:FABD2* after germination in paraquat (PQ)-containing media. (**A**–**D**) Root growth rate within the first 5 days after germination of the control C24 and *der1–3* mutant plants on the control media (**A**) and on media containing 0.1 (**B**), 0.2 (**C**) and 0.5 (**D**) µmol·L^−1^ of PQ. (**E**–**H**) Root growth rate within the first 5 days after germination of the transgenic C24 line carrying GFP-FABD2 and transgenic *der1–3* line carrying GFP-FABD2 on the control media (**E**) and on media containing 0.1 (**F**), 0.2 (**G**) and 0.5 (**H**) µmol·L^−1^ of PQ. (**I**–**L**) Average root growth per 24 h on the control media (**I**) and on media containing 0.1 (**J**), 0.2 (**K**) and 0.5 (**L**) µmol·L^−1^ of PQ. (**M**–**O**) Reduction ratio (fold change in respect to control) of average root growth in respective lines on media containing 0.1 (**M**), 0.2 (**N**) and 0.5 (**O**) µmol·L^−1^ of PQ. Experiments were repeated two times with 16 plants per line (control) and 12 plants per line (PQ). Different lowercase letters above the bars (**I**–**L**) represent statistical significance according to one-way ANOVA and subsequent LSD test at *p* value < 0.05.

In order to characterize the responses of the *der1–3* mutant to oxidative stress, we analyzed several phenotypical parameters. Apart from the obvious phenotype of root hairs that arrested their tip growth after bulge formation [42], mutant plants are also affected in other developmental aspects. Among them, seeds of the *der1–3* mutant germinate later than the C24 wild-type seeds, and primary roots show a more irregular and wavy growth pattern, due to obvious changes in the cell division plane orientation [45]. Analysis of the primary root growth of seedlings within the first 5 days after germination on the control media revealed a slightly lower elongation rate of the *der1–3* roots in comparison to the C24 (Figure 2A), however, the differences in average root growth per 24 h were insignificant (Figure 2I). We also found the same root growth rate in the transgenic *der1–3* mutant line carrying *pro35S::GFP:FABD2* (designated as *der1–3* GFP-FABD2; Figure 2I), but interestingly, the average root growth rate per 24 h of the transgenic C24 line carrying *pro35S::GFP:FABD2* (designated as C24 GFP-FABD2) on the control media was significantly higher (Figure 2E,I). Thus, seedlings of the transgenic C24 GFP-FABD2 line showed a more effective root growth rate than the C24 wild-type seedlings (Figure S1A), while there were no differences in this parameter between seedlings of the *der1–3* and transgenic *der1–3* GFP-FABD2 line in control conditions (Figure S1B).

Seedlings of all tested lines germinating and growing on PQ-containing media within the first 5 days after germination showed a reduction in primary root growth, which was dependent on PQ concentration (Figure S1C–F). Together with the flattening of the root growth rate curves, there was also the apparent PQ dose-dependent unification of the root growth rate between the C24 wild-type and *der1–3* mutant seedlings (Figure 2B–D), and also between the C24 GFP-FABD2 and *der1–3* GFP-FABD2 seedlings (Figure 2F–H). A dose-dependent reduction in average root growth per 24 h was apparent in seedlings germinating and growing on media containing 0.1, 0.2 and 0.5 µmol·L^−1^ of PQ (Figure 2J–L). Although the root growth rate of the *der1–3* mutant was always similar or lower in comparison to the C24 wild-type under control conditions (Figure 2A,I; Figure S1A,B), the roots of the *der1–3* mutant germinating and growing in the presence of 0.1 µmol·L^−1^ of PQ showed better growth than the C24 wild-type (Figure 2J). The average root growth rate per 24 h was considerably reduced on media containing 0.2 and 0.5 µmol·L^−1^ of PQ (Figure 2K,L) without any differences among all tested lines. However, when differences were evaluated as a reduction ratio in respect to control values, the fold change in average root growth rate on media containing 0.1 µmol·L^−1^ of PQ was 4.39 and 5.01 in the C24 wild-type and C24 GFP-FABD2, respectively, but only 2.93 and 3.10 in the *der1–3* mutant and *der1–3* GFP-FABD2, respectively (Figure 2M). Although the fold change in average root growth rate between the C24 and *der1–3* mutant genotypes were less obvious on media containing 0.2 and 0.5 µmol·L^−1^ of PQ, the reduction rate was similar or slightly lower in the *der1–3* mutant (Figure 2N,O). These data clearly indicate that the root growth of both the *der1–3* mutant and the *der1–3* GFP-FABD2 transgenic line is less affected by mild and severe oxidative stress induced by PQ presence in the culture medium.

The effectivity of the root growth rate under oxidative stress in analyzed lines was also determined by the measurement of the distance between the first root hair and the root tip. In roots of 5-day-old plants growing in control conditions, this distance was significantly longer in C24 wild-type in comparison to *der1–3* (Figure S2A). The same tendency showing a significantly longer distance between the first root hair and the root tip was also observed in the transgenic C24 GFP-FABD2 line in comparison to the transgenic *der1–3* GFP-FABD2 line (Figure S2A). Interestingly, both transgenic lines (C24 GFP-FABD2 and *der1–3* GFP-FABD2) had this measured distance significantly longer in comparison to the control C24 and *der1–3* plants, respectively (Figure S2A). Such differences between the C24 and *der1–3* mutant were reduced considerably, or disappeared completely, in seedlings germinating and growing on media containing 0.1, 0.2 and 0.5 µmol·L^−1^ of PQ (Figure S2B–D). This represented another indication of differential responses to oxidative stress of the analyzed lines, showing the significantly higher tolerance of the transgenic *der1–3* mutant line.

Plants monitored for 11 days after germination showed apparent time-dependent acceleration of root growth in control conditions (Figure S3A) with no obvious differences between the C24 wild-type and *der1–3* mutant (Figure S3E). However, root growth of the C24 GFP-FABD2 line was faster, particularly in the later stages of development (Figure S3A), leading to a significant increase in the average root growth per 24 h (Figure S3E). Monitoring root growth rate upon prolonged PQ treatment showed a clearly different trend of response between the control C24 lines and *der1–3* mutant lines. On media containing 0.1 and 0.2 µmol·L^−1^ of PQ, both the *der1–3* mutant and *der1–3* GFP-FABD2 line showed better average root growth per 24 h than the C24 wild-type and C24 GFP-FABD2 line (Figure S3F,G). Continuous monitoring of root growth rate revealed that it was higher in the *der1–3* mutant and *der1–3* GFP-FABD2 line than in the C24 wild-type and C24 GFP-FABD2 line from 8th to 11th day after germination (Figure S3B,C), and it was opposite to the control conditions (Figure S3A). The root growth of plants on media containing 0.5 µmol·L^−1^ of PQ was considerably reduced with minimal increase in length per day (Figure S3D), showing very similar average root growth per 24 h in all the examined lines (Figure S3H). This analysis confirmed that, unlike the wild-type lines, root growth and development of the *der1–3* mutant and *der1–3* GFP-FABD2 line is better adapted to the mild oxidative stress.

### 2.3. Biomass Production Affected by Oxidative Stress

Shoot and root fresh weights analyzed 18 days after germination of plants growing on the control media revealed a considerably higher biomass production in the C24 wild-type and C24 GFP-FABD2 line. Conversely, shoot and root biomass productions in the *der1–3* mutant and *der1–3* GFP-FABD2 line were seemingly lower (Figure S4A). In plants germinating and growing for 18 days on media containing PQ, both shoot and root biomass production declined, but not uniformly among the tested lines. While plants of the C24 wild-type and C24 GFP-FABD2 line reacted to increasing concentrations of PQ by drastic reduction of both shoot and root biomass, it was not so dramatically reduced in the *der1–3* mutant and *der1–3* GFP-FABD2 line. Already on media containing 0.1 and 0.2 µmol·L^−1^ of PQ (Figure S4B,C) biomass weights of the *der1–3* mutant and *der1–3* GFP-FABD2 line were similar or even higher as in C24 wild-type and C24 GFP-FABD2 line. Media with 0.5 µmol·L^−1^ of PQ massively hindered root development, but shoot biomass production and development was clearly better in *der1–3* mutant and *der1–3* GFP-FABD2 line (Figure S4D). Calculation of the biomass production as a reduction ratio in the fold change in respect to the control revealed minimal change in both shoot and root biomass in the *der1–3* mutant and *der1–3* GFP-FABD2 line on media containing 0.1 µmol·L^−1^ of PQ in comparison to the C24 wild-type and C24 GFP-FABD2 line (Figure S4E). On media containing 0.2 and 0.5 µmol·L^−1^ of PQ (Figure S4F,G), we found higher biomass production (in the fold change) in the *der1–3* mutant and *der1–3* GFP-FABD2 line when compared to the C24 wild-type and C24 GFP-FABD2 line. This analysis clearly revealed the physiological resistance of the *der1–3* mutant against mild and severe oxidative stress.

### 2.4. Plant Developmental Responses to Oxidative Stress

**Figure 3:**
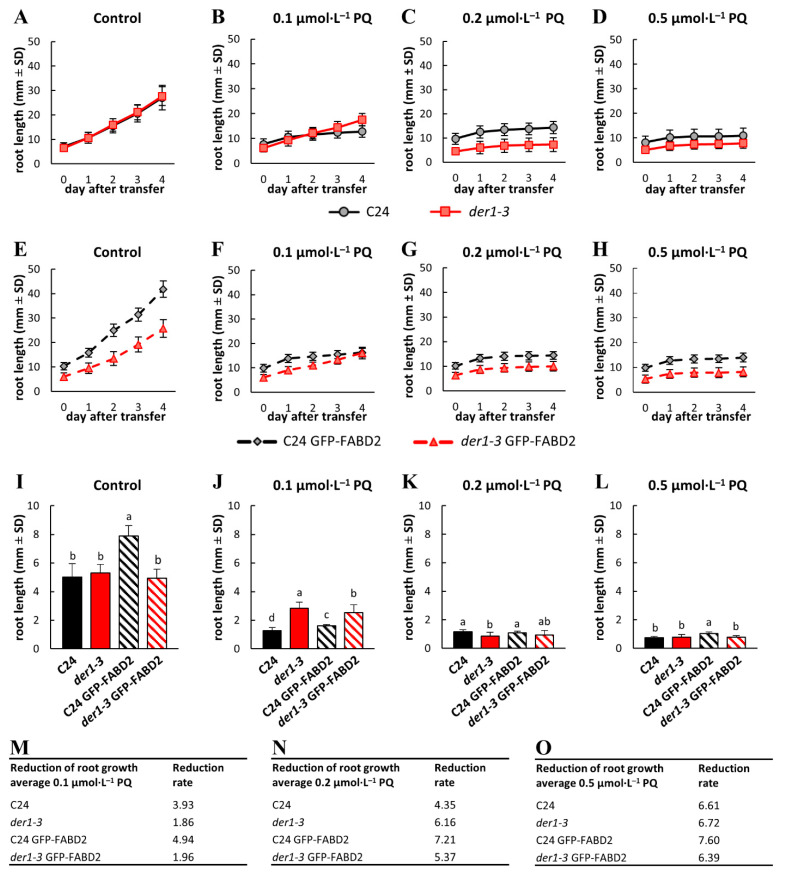
Root growth rate in plants of the control C24, *der1–3* mutant and transgenic C24 and *der1–3* lines expressing *pro35S::GFP:FABD2* after their transfer to PQ-containing media. 3-day-old plants germinated on the control media were transferred to PQ-containing media and the root growth rate was analyzed within the subsequent 4 days. (**A**–**D**) Root growth rate of the control C24 and *der1–3* mutant plants on the control media (**A**) and on media containing 0.1 (**B**), 0.2 (**C**) and 0.5 (**D**) µmol·L^−1^ of PQ. (**E**–**H**) Root growth rate of the transgenic C24 line carrying GFP-FABD2 and the transgenic *der1–3* line carrying GFP-FABD2 on the control media (**E**) and on media containing 0.1 (**F**), 0.2 (**G**) and 0.5 (**H**) µmol·L^−1^ of PQ. (**I**–**L**) Average root growth per 24 h on the control media (**I**) and on media containing 0.1 (**J**), 0.2 (**K**) and 0.5 (**L**) µmol·L^−1^ of PQ. (**M**–**O**) Reduction ratio (fold change in respect to control) of the average root growth in the respective lines on media containing 0.1 (**M**), 0.2 (**N**) and 0.5 (**O**) µmol·L^−1^ of PQ. Experiments were repeated two times with 16 plants per line (control) and 12 plants per line (PQ). Different lowercase letters above the bars (**I**–**L**) represent statistical significance according to one-way ANOVA and subsequent LSD test at *p* value < 0.05.

In order to characterize solely oxidative stress-related inhibition of root growth, we performed seed germination on the control media and after that, we transferred 3-day-old seedlings to a culture media containing different concentrations of PQ. Comparison of the root growth rate within 4 days after transfer showed that it is very similar for the C24 wild-type and *der1–3* mutant on the control media (Figure 3A,I). After the transfer of seedlings of transgenic lines, root growth rate on the control media of the C24 GFP-FABD2 line was significantly higher in comparison to the *der1–3* GFP-FABD2 line (Figure 3E). Actually, it was the highest among all tested lines (Figure 3I). Root growth rate of the C24 GFP-FABD2 line was higher as in C24 wild-type (Figure S5A), while there was no difference between the *der1–3* mutant and *der1–3* GFP-FABD2 line (Figure S5B). Transfer of the C24 wild-type and *der1–3* mutant seedlings germinated on the control media to media containing 0.1, 0.2 and 0.5 µmol·L^−1^ of PQ led to a similarly decreased root growth rate (Figure 3B–D). We also found a similar reaction in seedlings of the C24 GFP-FABD2 and *der1–3* GFP-FABD2 lines germinated on the control media and transferred to media containing the same concentrations of PQ (Figure 3F–H). Although the absolute root length of the *der1–3* mutant and *der1–3* GFP-FABD2 line was lower on media containing 0.2 and 0.5 µmol·L^−1^ of PQ (Figure 3C,D,G,H), the average root growth rate was not considerably different (Figure 3K,L). However, the reaction of seedlings to 0.1 µmol·L^−1^ of PQ revealed a much better tolerance of the *der1–3* mutant and *der1–3* GFP-FABD2 line, as their average root growth rate was significantly higher than in the C24 wild-type and C24 GFP-FABD2 line, respectively (Figure 3J). Different modes of seedling reaction after transfer to media with 0.1 µmol·L^−1^ of PQ was revealed. There was a rather uniform reduction of the root growth rate on PQ-containing media in the C24 wild-type and C24 GFP-FABD2 line (Figure S5C,D), while root growth rate much less affected by 0.1 µmol·L^−1^ of PQ was clearly documented in the *der1–3* mutant and *der1–3* GFP-FABD2 line (Figure S5E,F). These observations were corroborated by the quantitative characterization of differences in average root growth rate by a reduction ratio between the control and PQ-containing media in the fold changes. The root growth reduction ratio caused by 0.1 µmol·L^−1^ of PQ was lower in seedlings of the *der1–3* mutant and *der1–3* GFP-FABD2 line (Figure 3M). Using 0.2 µmol·L^−1^ of PQ, the differences between C24-related and *der1–3* mutant-related lines were lower in the *der1–3* GFP-FABD2 line, while the reduction was stronger in the *der1–3* mutant than in the C24 wild-type (Figure 3N). Differences between the lines transferred to 0.5 µmol·L^−1^ of PQ were negligible (Figure 3O).

**Figure 4:**
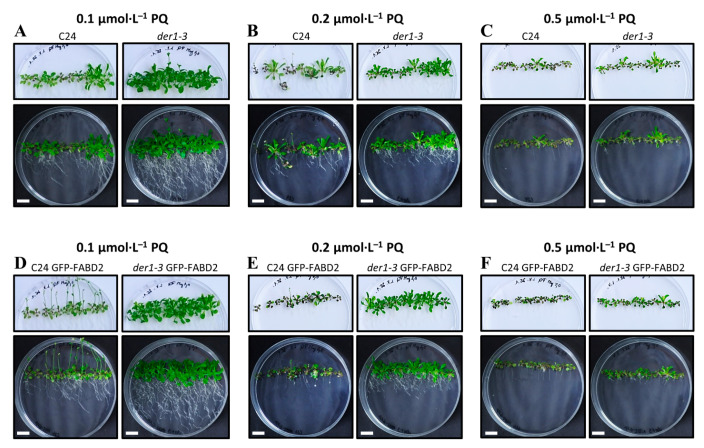
Plant phenotype of the control C24, *der1–3* mutant and transgenic C24 and *der1–3* lines expressing *pro35S::GFP:FABD2* after their transfer to PQ-containing media. 3-day-old plants germinated on the control media were transferred to PQ-containing media and photographed 17 days after transfer. (**A**–**C**) Plants of the control C24 and *der1–3* mutant growing on media containing 0.1 (**A**), 0.2 (**B**) and 0.5 (**C**) µmol·L^−1^ of PQ. (**D**–**F**) Plants of the transgenic C24 line carrying GFP-FABD2 and transgenic *der1–3* line carrying GFP-FABD2 growing on media containing 0.1 (**D**), 0.2 (**E**) and 0.5 (**F**) µmol·L^−1^ of PQ. Aboveground parts of plants were photographed on a white background (upper row of images), and whole plants including roots were documented on a black background (lower row of images). Plants grown on the control media are documented in Figure S6. Scale bar = 1 cm.

The phenotype of plants germinated and grown on the control media for 20 days confirmed smaller aboveground parts and a more irregular and wavy root growth pattern of the *der1–3* mutant (Figure S6A), in comparison to the C24 wild-type (Figure S6B). Transgenic plants of C24 GFP-FABD2 and *der1–3* GFP-FABD2 grown on the control media were bigger, but similar phenotypes, namely smaller aboveground parts and more irregular and wavy root growth pattern of *der1–3* GFP-FABD2 line, were still apparent (Figure S6C,D). However, plants transferred from the control to PQ-containing culture media revealed a better development of the *der1–3* mutant in comparison to the C24 wild-type (Figure 4A–C) and *der1–3* GFP-FABD2 line in comparison to the C24 GFP-FABD2 line (Figure 4D–F). In all concentrations of PQ tested, the aboveground parts of the *der1–3* mutant and *der1–3* GFP-FABD2 plants were much better developed (Figure 4). In addition, considering purple pigmentation and arrested leaf enlargement, plants of the *der1–3* mutant and *der1–3* GFP-FABD2 were much less affected in media containing 0.2 µmol·L^−1^ of PQ (Figure 4B,E) and 0.5 µmol·L^−1^ of PQ (Figure 4C,F). In media containing 0.1 µmol·L^−1^ of PQ, plants of the *der1–3* mutant and *der1–3* GFP-FABD2 line did not show such a strong stress reaction and developmental arrest (Figure 4A,D). Root development tested at the same conditions showed a clear genotype-dependent response to PQ-induced oxidative stress. Plants (3 days-old) transferred from control to PQ-containing plates and photographed 17 days after transfer showed that the *der1–3* mutant and *der1–3* GFP-FABD2 line are much less sensitive to 0.1 µmol·L^−1^ of PQ than the C24 wild-type and C24 GFP-FABD2 line, respectively (Figure 4A,D). The root system of the *der1–3* mutant (Figure 4A) and *der1–3* GFP-FABD2 line (Figure 4D) maintained the ability to grow and develop. Although 0.2 µmol·L^−1^ of PQ considerably reduced root development, the growing and branching capacity of the *der1–3* mutant (Figure 4B) and *der1–3* GFP-FABD2 line (Figure 4E) were higher in comparison to the C24 wild-type and C24 GFP-FABD2 line. Adding 0.5 µmol·L^−1^ of PQ to the culture medium dramatically reduced the root development of all the tested lines (Figure 4C,F), which was also apparent from the root growth rate (Figure 3L) and root fresh weight (Figure S4D) analyses. Putting together analyses of post-germination root growth and plant development after transfer to PQ-containing media from control conditions confirmed that plants of the *der1–3* mutant and *der1–3* GFP-FABD2 line are more tolerant, particularly to the mild oxidative stress.

**Figure 5:**
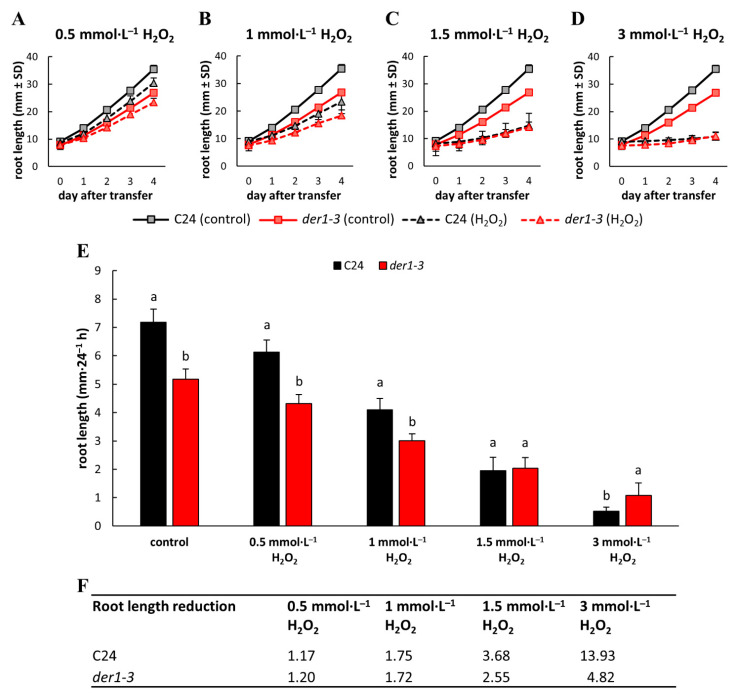
Root growth rate of the control C24 and *der1–3* mutant plants after their transfer to H_2_O_2_-containing media. 3-day-old plants germinated on the control media were transferred to a H_2_O_2_-containing media and root growth rate was analyzed within the subsequent 4 days. (**A**–**D**) Root growth rate of the control C24 and *der1–3* mutant plants on media containing 0.5 (**A**), 1 (**B**), 1.5 (**C**) and 3 (**D**) mmol·L^−1^ of H_2_O_2_. (**E**) Average root growth per 24 h on the control media and on media containing indicated concentrations of H_2_O_2_. (**F**) Reduction ratio (fold change in respect to control) of average root growth in the control C24 and *der1–3* mutant plants on media containing 0.5 (**A**), 1 (**B**), 1.5 (**C**) and 3 (**D**) mmol·L^−1^ of H_2_O_2_. Experiments were repeated two times with 10 plants per line. Different lowercase letters above the bars (**E**) represent statistical significance according to one-way ANOVA and subsequent LSD test at *p* value < 0.05.

Taking into account the inhibitory effects of PQ in photosynthetically active plant tissues, we also employed a H_2_O_2_ treatment, as an alternative oxidative stress-inducing agent that directly affects the root system and its development. Four different concentrations of H_2_O_2_ (0.5; 1; 1.5 and 3 mmol·L^−1^) were tested in post-germination root growth rate analysis within 4 days after the transfer of 3-day-old seedlings germinated on the control media. We observed a H_2_O_2_ dose-dependent response in the inhibition of root elongation (Figure 5A–D). The average root length of both compared lines was gradually reduced by the presence of 0.5, 1, 1.5 and 3 mmol·L^−1^ of H_2_O_2_ (Figure 5A–E) in the culture medium. We observed significantly longer roots of the C24 wild-type plants than the *der1–3* mutant plants within the testing period in control conditions, and on media containing 0.5 and 1 mmol·L^−1^ of H_2_O_2_, however, there was no statistically significant difference in the root length on media containing 1.5 mmol·L^−1^ of H_2_O_2_ (Figure 5E). Interestingly, the stronger concentration of H_2_O_2_ (3 mmol·L^−1^) inhibited root elongation in the C24 wild-type significantly more than in the *der1–3* mutant plants (Figure 5E).

The quantitative characterization of differences in the average root growth rate presented as a reduction ratio between the control and H_2_O_2_-containing media showed no differences between the C24 wild-type and *der1–3* mutant on media containing 0.5 and 1 mmol·L^−1^ of H_2_O_2_ (Figure 5F). However, a moderate difference caused by 1.5 mmol·L^−1^ of H_2_O_2_ and a considerably increased difference induced by 3 mmol·L^−1^ of H_2_O_2_ (Figure 5F) suggested that root growth and development of the *der1–3* mutant plants are substantially more resistant to moderate and severe oxidative stress than of the C24 wild-type plants. It can be further documented also by the phenotype of whole plants. Together with the root system that was severely reduced by increasing the concentration of H_2_O_2_ in the C24 wild-type plants, a reduction in the development of their aboveground parts was also obvious (Figure S7A). In comparison, although the development of the root system of the *der1–3* mutant plants was also reduced by increasing the concentration of H_2_O_2_, the development of their aboveground parts was less affected (Figure S7B). The overall data of phenotypical analyses thus indicate that the *der1–3* mutant and transgenic plants in the *der1–3* mutant background maintain growth and development because they are better protected against PQ- or H_2_O_2_-induced oxidative stress.

### 2.5. Oxidative Stress and Response of the Actin Cytoskeleton

**Figure 6:**
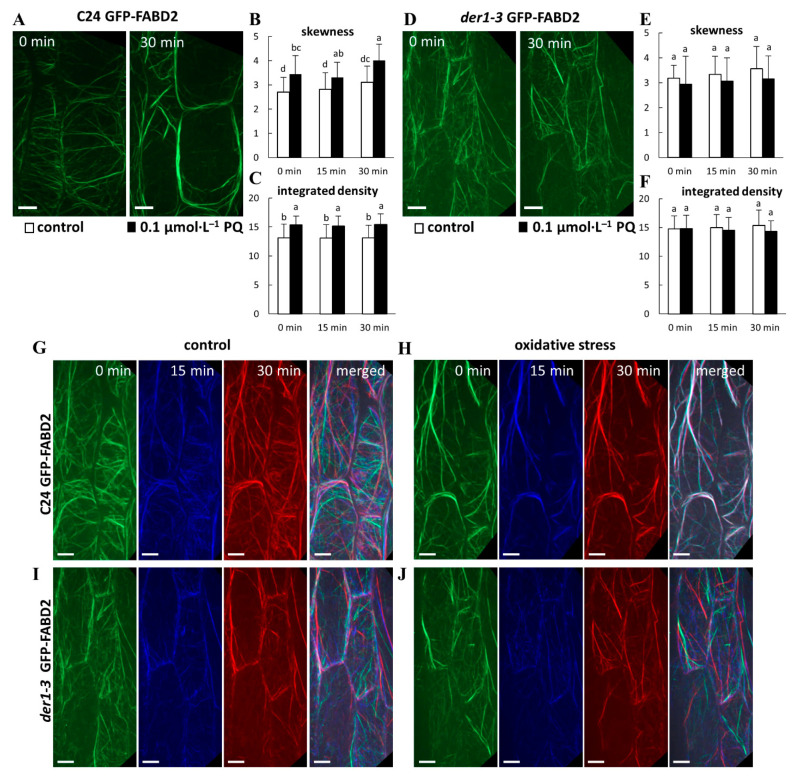
Organization and dynamics of actin filaments in the hypocotyl epidermal cells of transgenic C24 and *der1–3* lines expressing *pro35S::GFP:FABD2* under PQ-induced oxidative stress. (**A**–**C**) Actin filaments in hypocotyl epidermal cells of 3-day-old plants of the C24 GFP-FABD2 line in control conditions and after treatment with 0.1 µmol·L^−1^ of PQ for 30 min (**A**). Quantitative analysis of the actin filament bundling extent (skewness, **B**) and the actin filament density (percentage of occupancy, **C**) in control conditions and after the application of 0.1 µmol·L^−1^ of PQ. (**D**–**F**) Actin filaments in the hypocotyl epidermal cells of 3-day-old plants of the *der1–3* GFP-FABD2 line in control conditions and after treatment with 0.1 µmol·L^−1^ of PQ for 30 min (**D**). Quantitative analysis of the actin filament bundling extent (skewness, **E**) and the actin filament density (percentage of occupancy, **F**) in control conditions and after the application of 0.1 µmol·L^−1^ of PQ. Data were analyzed on images collected from hypocotyl epidermal cells within 0, 15 and 30 min time-points of scanning. (**G**–**J**) Semiquantitative analysis of actin filament dynamics in hypocotyl epidermal cells presented by pseudocoloring displacement analysis. Dynamic properties of actin filaments in the C24 GFP-FABD2 line in control conditions (**G**) and after the application of 0.1 µmol·L^−1^ of PQ (**H**). Dynamic properties of the actin filaments in the *der1–3* GFP-FABD2 line in control conditions (**I**) and after the application of 0.1 µmol·L^−1^ of PQ (**J**). Images acquired at the beginning, after 15 min and after 30 min of time-point scanning were colored red, green, and blue, respectively, and merged. A white color indicates lowering (eventually stopping) of the actin dynamic activity. Experiments were repeated 5–6 times with 4–5 cells per plant in each line. Different lowercase letters above the bars (**B**,**C**,**E**,**F**) represent statistical significance according to one-way ANOVA and subsequent LSD test at *p* value < 0.05. Scale bar = 10 µm.

In order to characterize the organization and dynamic properties of the actin cytoskeleton under PQ-induced oxidative stress, we utilized transgenic C24 and *der1–3* lines expressing *pro35S::GFP:FABD2* construct. In hypocotyl epidermal cells of 3-day-old plants of the C24 GFP-FABD2 line in control conditions, actin filaments were arranged in an extensive, well-organized and dynamic network (Video S3). However, we observed massive bundling, particularly in cortical layers of the cell after treatment with 0.1 µmol·L^−1^ of PQ for 30 min (Figure 6A; Video S4). The semi-quantitative evaluation of actin filament skewness, determining a degree of actin filament bundling, showed increased values after application of oxidative stress (Figure 6B). The semi-quantitative evaluation of integrated density, determining fluorescence signal intensity per 1 µm^2^, was also significantly increased after PQ treatment (Figure 6C). Actin filament organization was slightly different in hypocotyl epidermal cells of 3-day-old *der1–3* GFP-FABD2 plants in control conditions, showing mainly thinner, less organized, but dynamic actin filaments in cell cortex (Video S5). Treatment with 0.1 µmol·L^−1^ of PQ for 30 min induced partial bundling of actin filaments, but overall changes in their organization and dynamics were not so dramatic (Figure 6D; Video S6). As a result, both actin filament skewness (Figure 6E) and integrated density, determining mean fluorescence signal intensity (Figure 6F), were not significantly affected by oxidative stress.

Next, dynamic properties of the actin cytoskeleton in hypocotyl epidermal cells were analyzed by sequential imaging of actin filaments within 30 min (by acquiring 0, 15 and 30 min time-points), followed by pseudocolor–based evaluation of their lateral displacement. In control conditions, dynamic changes of the actin filament network in cells of both C24 GFP-FABD2 (Figure 6G; Video S3) and *der1–3* GFP-FABD2 (Figure 6I; Video S5) lines were determined by the minimal overlay of sequential colored scans in merged images. After the application of 0.1 µmol·L^−1^ of PQ, the same analysis revealed the formation of excessive actin bundles with minimal dynamic changes in structure and organization in cells of the C24 GFP-FABD2 line (Figure 6H; Video S4), while far fewer bundles were formed in cells of the *der1–3* GFP-FABD2 line. In addition, the overlay of sequential colored scans revealed unchanged dynamic properties that were still high, particularly in fine actin filaments (Figure 6J; Video S6). This analysis showing alterations in structure and dynamic properties of the actin cytoskeleton in the *der1–3* GFP-FABD2 line, that were not considerably affected by PQ treatment, may significantly support observed physiological resistance of the *der1–3* mutant and the related transgenic *der1–3* GFP-FABD2 line against oxidative stress.

### 2.6. ROS Production, Lipid Peroxidation and Antioxidant Activity

The relative levels of ROS were determined by the histochemical ROS detection in the cotyledons and leaves of seedlings under control and oxidative stress-inducing conditions. Visualization was performed using NBT (nitrotetrazolium blue) staining of O_2_^•−^ production and DAB (diaminobenzidine) staining of H_2_O_2_ production, respectively. Semi-quantitative evaluation of the mean staining intensity revealed that both in cotyledons (Figure S8A) and leaves (Figure S8B) of plants treated for 7 days, there was no difference in O_2_^•−^ production upon PQ and H_2_O_2_ treatments between the C24 wild-type and the *der1–3* mutant. The ability of H_2_O_2_ production visualized by DAB staining was lower in the *der1–3* mutant than in the C24 wild-type in control conditions, both in cotyledons (Figure S8C) and leaves (Figure S8D). Interestingly, upon PQ and H_2_O_2_ treatments, the level of H_2_O_2_ production in the *der1–3* mutant was increased to the C24 wild-type level, both in cotyledons and leaves (Figure S8C,D).

**Figure 7:**
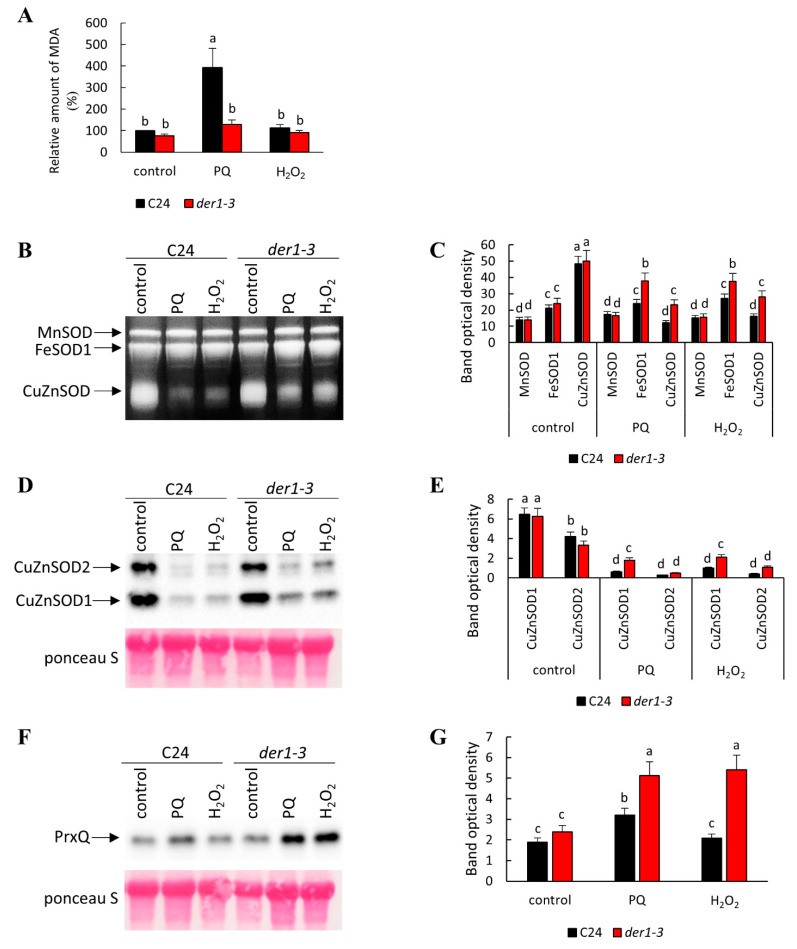
Estimation of lipid peroxidation and antioxidant capacity in plants of C24 wild-type and *der1–3* mutant. 3-day-old plants germinated on the control media were transferred to 0.1 µmol·L^−1^ of PQ- and 3 mmol·L^−1^ of H_2_O_2_-containing media and biochemical analysis was performed 11 days after the transfer (plants were 14 days old). (**A**) Relative quantification of malondialdehyde content. (**B**,**C**) Visualization of superoxide dismutase (SOD) isoforms activity (**B**) and quantification of individual band densities (**C**) on native polyacrylamide gels. (**D**,**E)** Immunoblot of CuZnSOD1 and CuZnSOD2 isoforms (**D**) and quantification of band densities (**E)**. (**F**,**G**) Immunoblot of peroxiredoxin Q abundance (**F**) and quantification of band densities (**G**). Different lowercase letters above the bars (**A**,**C**,**E**,**G**) represent statistical significance between treatments according to *t*-Test at *p* value < 0.05. Uncropped, full original images of immunoblots are provided in Figures S9 and S10.

Based on observed phenotypical differences, we aimed to provide evidence about the biochemical mechanisms underlying an increased tolerance of the *der1–3* mutant to oxidative stress. Our analyses showed that the *der1–3* mutant exhibited a lower degree of lipid peroxidation after long-term PQ treatment compared to the C24 wild-type, while cultivation on H_2_O_2_-containing media did not cause lipid peroxidation in any examined line (Figure 7A). This indicated that PQ treatment was less damaging to the *der1–3* mutant compared to C24 in terms of membrane integrity. Next, we also examined activities of important antioxidant enzymes in the *der1–3* mutant and C24 wild-type. We found the elevated capacity to decompose O_2_^•−^ in the *der1–3* mutant, as manifested by a more intensive activation of iron superoxide dismutase 1 (FeSOD1) in these plants when exposed to both PQ and H_2_O_2_, compared to the C24 wild-type (Figure 7B,C). Both treatments substantially decreased the activity of copper-zinc superoxide dismutase (CuZnSOD) isoforms in both the *der1–3* mutant and the C24 wild-type, while this reduction was less pronounced in the *der1–3* mutant (Figure 7B,C). This was also observed on protein abundance levels, as shown by immunoblotting (Figure 7D,E). In addition, we also encountered a substantially increased abundance of chloroplastic PEROXIREDOXIN Q (PrxQ), a H_2_O_2_ decomposing enzyme, in the *der1–3* mutant in response to both PQ and H_2_O_2_ treatments, while a slight increase (in the case of PQ), or unchanged abundance (in the case of H_2_O_2_), were observed in the C24 wild-type (Figure 7F,G). Altogether, these results showed that an increased tolerance of the *der1–3* mutant plants to oxidative stress is determined by an elevated enzymatic capacity to decompose ROS.

**Figure 8:**
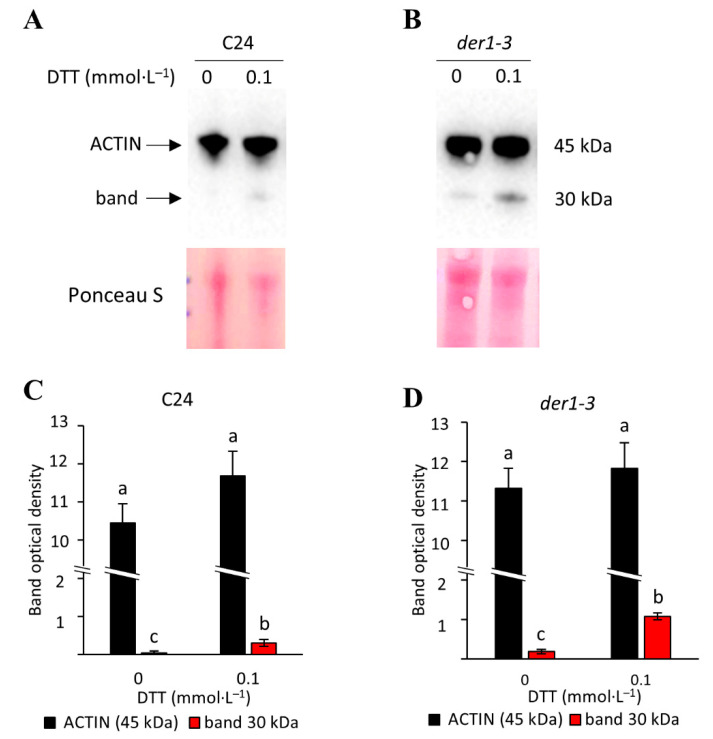
Examination of ACTIN sensitivity to dithiothreitol (DTT) in the C24 wild-type and *der1–3* mutant. (**A**–**B**) Immunoblots of ACTIN prepared by using anti-ACTIN 2, 8, 11 primary antibody in the C24 wild-type (**A**) and *der1–3* mutant (**B**). (**C**,**D**) Quantification of band densities in (**A**,**B**), respectively. Different lowercase letters above the bars (**C**,**D**) represent statistical significance between treatments according to *t*-Test at *p* value < 0.05. Uncropped, full original images of immunoblots are provided in Figure S11.

The altered ROS metabolism in the *der1–3* mutant implies that point mutation exchanging Arg97 to Cys97 makes ACTIN2 in the *der1–3* mutant more prone to oxidative modifications. This might be supported also by the fact that Cys is one of the two amino acids undergoing oxidative posttranslational modifications. To test this, we monitored ACTIN protein(s) using extraction in the presence of the reducing agent dithiothreitol (DTT) and immunoblots on C24 and the *der1–3* mutant. We found that 0.1 mmol·L^−1^ of DTT induced an additional band of 30 kDa along with the specific band (45 kDa) corresponding to the ACTIN 2, 8 and 11 isoforms recognized by the specific monoclonal anti-ACTIN antibody (Figure 8A,B). Considering that ACTIN 11 is expressed only in reproductive organs, the immunoreactive bands (Figure 8A,B) likely reflect the relative abundance of ACTIN 2 and ACTIN 8 isoforms. The 30 kDa band showed higher DTT-induced abundance in the *der1–3* mutant (Figure 8D) compared to the C24 line (Figure 8C). Thus, ACTIN in the *der1–3* mutant shows increased sensitivity to DTT, indicating an amended capacity for redox modifications.

## 3. Discussion

The random mutagenesis approach in *Arabidopsis thaliana* led to the isolation of *der1* mutants, bearing a single-point mutation in the *DER1* locus. It was demonstrated that these mutants (*der1–1*, *der1–2*, *der1–3*) are compromised in root hair development after the bulge initiation, showing typical “short root hair” phenotypes [42]. The map-based cloning of the *DER1* locus revealed mutation in the *ACTIN2* gene [42]. Locations of these mutations to the *ACTIN2* confirmed the essential role of the actin cytoskeleton in the root hair development [42,46]. Further phenotypical, developmental and microscopic analyses of *der1–3* mutant plants revealed that due to changes in the cell division plane orientation, primary roots showed an irregular and wavy pattern while actin filaments in epidermal cells of different plant organs (roots, hypocotyls and cotyledons) were shorter, thinner and arranged in more random orientations [45]. Thus, the *der1–3* mutant was affected in a broader range of morphological and developmental aspects, related to alterations of the actin cytoskeleton and its organization at a cellular level. It is not clear how structural and dynamic properties of the actin cytoskeleton may support plant reactions to oxidative stress, therefore we addressed this in the present study. We induced conditions of mild and severe oxidative stress by supplementing PQ or H_2_O_2_ to the culture medium and characterized diverse parameters such as root growth, development and biomass production in the *der1–3* mutant and C24 wild-type plants. Analyses were performed on plants germinating directly on such oxidative stress-inducing media or on seedlings germinated on the control media first and subsequently transferred to PQ- or H_2_O_2_-containing plates.

We found that oxidative stress induced by three different concentrations of PQ has a clear negative effect on the root growth of the C24 plants and *der1–3* mutant. We prepared transgenic lines of C24 and *der1–3* expressing *pro35S::GFP:FABD2* construct in order to perform live-cell microscopic characterization of the actin cytoskeleton, its organization and dynamic properties in these lines. We also characterized the root growth rate in all above-mentioned lines. Interestingly, root growth of both the *der1–3* mutant and the *der1–3* GFP-FABD2 transgenic line was much less affected by mild and severe PQ-induced oxidative stress as in the control C24, particularly at the concentration 0.1 µmol·L^−1^ of PQ in the culture medium. Consequently, the reduction ratio in average root growth quantified as a fold change in respect to the C24 control was several orders lower in the *der1–3* mutant (Figure 2M, Figure 3M and Figure 5F). We found a similar tendency in the reduction of biomass production by the PQ treatment, which was several orders stronger in the C24 wild-type than in the *der1–3* mutant. This trend was documented in both root and shoot biomass production and recorded in all PQ concentrations tested (Figure S4E–G). All data from analyses of post-germination root growth and plant development, both germinating on PQ-containing media and after the transfer of non-treated seedlings to PQ-containing media, indicate that plants of the *der1–3* mutant and *der1–3* GFP-FABD2 line are more tolerant, particularly to the mild oxidative stress.

The observed changes of phenotypical parameters distinguishing the C24 wild-type from the *der1–3* mutant indicate different sensitivities to oxidative stress. In addition, we performed several supporting biochemical experiments. The estimation of lipid peroxidation based on the relative quantification of malondialdehyde content revealed that the *der1–3* mutant exhibits a lower degree of lipid peroxidation after long-term PQ treatment compared to the C24 wild-type. Thus, an important aspect of antioxidant defense in plants, namely membrane integrity, was better protected in the *der1–3* mutant. Better tolerance of the *der1–3* mutant against oxidative stress was also supported by the abundance and activity of antioxidant enzymes such as iron superoxide dismutase 1 (FeSOD1) and two copper-zinc superoxide dismutase isoforms (CuZnSOD1 and CuZnSOD2). Elevated levels of FeSOD1 (activity) and CuZnSOD1/2 (activity and abundance) in the *der1–3* mutant after long-term PQ and H_2_O_2_ exposure point to the higher capacity of the mutant to decompose O_2_^•−^ compared to the C24 wild-type. CuZnSOD1/2 and FeSOD1 are proposed as important determinants of oxidative stress tolerance [47,48]. The *der1–3* mutant also showed increased H_2_O_2_ decomposing efficiency which is executed by PrxQ. Nevertheless, other mechanisms of H_2_O_2_ removal cannot be excluded. PrxQ is an atypical 2-cys peroxiredoxin which uses (and interacts with) thioredoxin as an electron donor to decompose H_2_O_2_ [49,50]. Peroxiredoxins and thioredoxins as redox buffering proteins, may also modulate intracellular signaling related to ROS [51]. Thus, we propose that the higher capacity to decompose ROS and enhanced cellular redox regulation might represent main factors determining an increased tolerance of the *der1–3* mutant to oxidative stress.

The next task was to reveal how the structure and organization of the actin cytoskeleton in *der1–3* may support increased tolerance of this mutant to oxidative stress. Previous studies reported that the *der1–3* mutant does not show solely root hair phenotype, but the actin cytoskeleton was altered and also affected root growth and development. Actin filaments in cells of the *der1–3* mutant were shorter, thinner and arranged in more random orientations [45]. Oxidative stress caused by application of 0.1 µmol·L^−1^ of PQ for 30 min induced massive bundling of actin filaments in cells of the C24 GFP-FABD2 line. The actin cytoskeleton in cells of the *der1–3* GFP-FABD2 line was arranged in an extensive network, although actin filaments were thinner and less organized. However, this organization was virtually insensitive to the 0.1 µmol·L^−1^ of PQ applied for 30 min. A higher protection of the actin fine network in the *der1–3* GFP-FABD2 line was accompanied by roughly unchanged dynamic properties under PQ treatment. Thus, higher resistance of the actin cytoskeleton against the deteriorating effects of oxidative stress may be one of the main molecular mechanisms supporting the higher tolerance of the *der1–3* mutant to this type of stress. This can be related to the proposed role of the actin cytoskeleton in the adaptation of Arabidopsis root meristem cells to oxidative stress through protecting PIN2 auxin efflux carrier trafficking to the plasma membrane, which is controlled by auxin levels. Since auxin levels were disturbed by generated ROS, the abundance of PIN2 at the plasma membrane decreased. The role of the actin cytoskeleton lies on keeping the PIN2 intracellular trafficking, which requires the function of the ADP-ribosylation factor (ARF)-guaninenucleotide exchange factor (GEF) BEN1, an actin-associated regulator [39]. However, it is not known how this, and similar functions can be affected by altered structural and dynamic properties of the actin cytoskeleton in the *der1–3* mutant. It was proposed that PIN2 intracellular trafficking was reduced because H_2_O_2_ treatment affected actin dynamics [39]. A reduction in actin filament bundling can be directly associated with increased actin filament dynamics [34]. Similarly, the treatment of Arabidopsis plants with strigolactones reduces bundling of actin filaments with their simultaneously increasing dynamics, however, *der1–2* and *der1–3* mutants were much less sensitive to strigolactone analogue GR24 [52]. Collectively, these data support our conclusion that the actin filament arrangement is less prone to bundling and staying dynamic is critical for actin properties in the *der1–3* mutant, significantly contributing also to higher tolerance of this mutant against oxidative stress.

The position of the mutated amino acid Arg-97 in the ACTIN2 sequence is located in the subdomain 1 on the protein surface ([42,53,54]; Figure 1A; Video S1). The topology of this modification might have an impact on the biochemical properties of ACTIN2 in the *der1–3* mutant. There are some supporting facts for this. BEN1, a guanine exchange factor for ARF, regulating the actin filament-based intracellular trafficking of PIN2 during adaptation to oxidative stress, contains highly conserved cysteine residues [39,55] that could be modified by H_2_O_2_ treatment. Increased redox status upon the accumulation of H_2_O_2_ can initiate the oxidation of cysteine sulfhydryl groups in actins [56]. As mutated ACTIN2 protein in the *der1–3* mutant contains additional Cys compared to the native one, we hypothesize that ACTIN2 in *der1–3* might undergo redox-mediated posttranslational modifications accelerating the antioxidant capacity in the *der1–3* mutant via PrxQ and thioredoxins.

The actin cytoskeleton is recognized as a part of the plant stress sensing machinery and contributes to abiotic and biotic stress responses [57]. For example, actin depolymerization induces the salicylic acid signaling pathway [58], also having an impact on the abundance of proteins involved in abscisic acid signaling [59]. The actin nucleating complex containing actin-related proteins 2 and 3 (ARP2/3) is necessary for H_2_O_2_-dependent stomatal closure in Arabidopsis [60]. During elicitation by diverse plant pathogen elicitors, the actin cytoskeleton is rapidly remodeled through the activation of respiratory burst oxidase homolog protein D (RbohD)-dependent ROS production. This ROS signal is transduced to the actin cytoskeleton by heterodimeric capping protein [61]. In addition, there is evidence about direct oxidative posttranslational modifications of ACTIN in mammalian cells, including oxidation [62], glutathionylation [63] or S-sulfhydration [64]. These modifications affect ACTIN depolymerization [62] and dynamics [65,66]. In this study, we provide biochemical evidence that ACTIN might be more prone to undergo redox posttranslational modifications in the *der1–3* mutant. Redox modification by DTT resulted in higher abundance of a band with 30 kDa, which resembles an actin fragment produced by actin cleavage in mammalian cells [67,68]. Thus, our results indicate that ACTIN redox modifications might influence its biochemical properties, which is likely associated with the modified oxidative stress response in the *der1–3* mutant.

Putting together, our data indicate that a topologically important change in ACTIN2 in the *der1–3* mutant is linked to a better tolerance to mild and severe oxidative stress, increased capacity to decompose ROS and higher dynamicity of the actin cytoskeleton.

## 4. Materials and Methods

### 4.1. Plant Material and Cultivation In Vitro

Seeds of *Arabidopsis thaliana* (L.) Heynh., ecotype C24, *der1–3* mutant (kindly provided by Christoph Ringli, [42]) and transgenic lines expressing markers for visualization of the actin cytoskeleton were surface sterilized and planted into ½ Murasighe and Skoog medium without vitamins solidified with 0.6% (*w*/*v*) Gellan gum (Alfa Aesar, ThermoFisher Scientific, Waltham, MA, USA). Seeds on medium in Petri dishes were stratified at 4 °C for 3 days for synchronized germination. After stratification, seeds on plates were cultivated *in vitro* vertically in a culture chamber at 21 °C, 70% humidity, and 16/8h light/dark cycle. The level of photosynthetically active radiation (PAR) source was 120 μmol·m^−2^·s^−1^ (400–700 nm), provided by cool white fluorescent linear tube light sources (Philips Master TL-D Reflex 36W, light flow 3350 lm, light efficiency 93 lm·W^−1^).

### 4.2. Transgenic Lines and Transformation Method

Plants of *Arabidopsis thaliana* (L.) Heynh. ecotype C24 [69] and the *der1–3* mutant [42] were transformed with *Agrobacterium tumefaciens* stain GV3101 carrying a construct *pro35S::GFP:FABD2*, coding for F-actin binding domain 2 of Arabidopsis FIMBRIN 1 (FABD2) fused to green fluorescent protein (GFP); [70]. These lines were used for fluorescent visualization of actin filaments [45]. Briefly, this construct was prepared in a pCB302 vector with rifampicin and kanamycin resistance by the classical cloning method with herbicide phosphinothricin as the selection marker *in planta*. Stable transformation was used according to [71]. Plants (3–4 weeks old) were soaked in *Agrobacterium tumefaciens* cultures for 10 s and were stabilized in the dark overnight. After that, plants were cultivated in a culture chamber at 24 °C, 60% humidity, 16/8 h light/dark photoperiod. Transformation was repeated after one week. Seeds of T_1_ generation were planted for selection on ½ MS media with phosphinothricin (50 mg·mL^−1^). Transgenic plants were selected for the presence of GFP fusion proteins using an epifluorescence zoom microscope Axio Zoom.V16 (Carl Zeiss, Germany). For further experiments, seeds of T_3_ generation were used.

### 4.3. Application of Stress Factors

Oxidative stress was induced by adding three different concentrations of PQ (0.1; 0.2 and 0.5 µmol·L^−1^), and four different concentrations of H_2_O_2_ (0.5; 1; 1.5 and 3 mmol·L^−1^) to the culture medium. Either seeds were planted directly on ½ MS media containing different concentrations of PQ, or 3-day-old plants germinated on the control media were transferred to media containing different concentrations of PQ or H_2_O_2_.

### 4.4. Phenotypical Analysis

Plants germinating and growing *in vitro* on the control media, or on media containing different concentrations of PQ, were scanned directly on plates every 24 h for 11 days from the day of germination. Plants germinating on the control media and transferred to stress conditions were scanned on plates every 24 h for an additional 4 days after their transfer. Images from the scanner (Image Scanner III, GE Healthcare, Chicago, IL, USA) were used for the measurement of the primary root length. Images documenting the phenotype of plants growing in plates were prepared with a Nikon 7000 camera equipped with a macro-objective Sigma 50 mm (2.8 focal distance) in time points indicated in the corresponding figure captions. Fresh weights of separated shoots and roots were measured from 18-day-old plants growing on media containing PQ.

### 4.5. Sample Preparation and Microscopic Analysis

Samples for microscopic analysis were prepared in microscopic chambers filled with the liquid culture medium according to [72]. Oxidative stress was induced using the liquid culture medium containing 0.1 µmol·L^−1^ of PQ. Samples were firstly observed under the microscope in the control medium for 30 min and then the medium containing PQ was applied using perfusion of the microscopic chamber. The total volume of the medium applied was 100 µL, added by perfusion sequentially 10 times with 10 µL. After perfusion, hypocotyl and cotyledon above the coverslip was carefully covered with parafilm and samples were scanned in the microscope every 30 s for a further 30 min. Live cell imaging of the actin cytoskeleton in hypocotyl epidermal cells of the C24 ecotype and *der1–3* mutant expressing a construct *pro35S::GFP:FABD2* was performed in a fast scanning mode using a spinning disk microscope Cell Observer SD Axio Observer Z1 (Carl Zeiss, Germany), equipped with EC Plan-Neofluar 40×/1.3 NA oil immersion objective (Carl Zeiss, Germany) and Plan-Apochromat 63×/1.4 NA oil immersion objective (Carl Zeiss, Germany). Samples were imaged with an excitation laser line 488 nm and emission filter BP525/50. Laser power was set up not to exceed 50% of the laser intensity range available. Samples were scanned in a Z-stack mode in a time range of every 30 s for 30 min. Images were acquired with the Evolve 512 EM CCD camera with the exposure time 500–750 ms per optical section. Orthogonal projections of 6 to 10 optical sections from Z-stacks were used for the preparation of videos and measurement of actin filament skewness and occupancy. Semiquantitative analysis of actin filament dynamics in hypocotyl epidermal cells was presented by pseudocoloring displacement analysis. Images were acquired at the beginning, after 15 min and after 30 min of time-point scanning, individually colored red, green, and blue, respectively, and merged. Overlay of all three colors creating a white one indicated lowering, or eventually stopping, of the actin dynamic activity.

### 4.6. Histochemical Detection of O_2_^•−^ and H_2_O_2_ Production

Plants (3 days old) were transferred from the control media to media containing 0.1 µmol·L^−1^ of PQ and 3 mmol·L^−1^ of H_2_O_2_ and histochemical detection of ROS was performed 11 days after the transfer (plants were 14 days old). Superoxide (O_2_^•−^) was detected by NBT (nitrotetrazolium blue) staining according to [73]. H_2_O_2_ detection was performed with DAB (diaminobenzidine) staining according to [74]. After staining, plants were mounted and imaged in Axio Zoom.V16 (Carl Zeiss, Germany). Staining intensity mean values in cotyledons and leaves were measured and quantified in ZEN 2 (blue edition; Carl Zeiss, Germany) software.

### 4.7. Analysis of Enzymatic Activity and Immunoblotting

The plant material for enzyme analyses was prepared as described in Section 4.6. For superoxide dismutase (SOD) activity examination, proteins were extracted using a Na-phosphate extraction buffer containing 50 mmol·L^−1^ of Na-phosphate buffer (pH 7.8), 2 mmol·L^−1^ of EDTA, 2 mmol·L^−1^ of ascorbic acid and 10% (*v*/*v*) glycerol. SOD activities were visualized on native PAGE gels as described in [75]. For the immunoblotting of CSD and PrxQ, the enzyme extracts were enriched with a 4× Laemmli SDS buffer (to reach a final concentration of 10% (*v*/*v*) glycerol, 60 mmol·L^−1^ of Tris/HCl pH 6.8, 2% (*w*/*v*) SDS, 0.002% (*w*/*v*) bromophenol blue and 5% (*v*/*v*) β-mercaptoethanol). Afterwards, the samples were boiled at 95 °C for 5 min. To evaluate the response of ACTIN to DTT, proteins were extracted from 14 day-old C24 wild-type and *der1–3* plants ground to fine powder using liquid nitrogen as described in [76] with modifications. Homogenates were incubated for 1 h at RT in 62.5 mmol·L^−1^ of Tris-HCl buffer (pH 6.8) supplemented with 2% (*w*/*v*) SDS, 7.5% (*v*/*v*) glycerol, 0.01% (*w*/*v*) bromophenol blue, complete EDTA-free protease inhibitor cocktail (Roche Life Science, Penzberg, Germany), and without or with 0.1 mmol·L^−1^ of DTT. Subsequently, the extracts were boiled at 95 °C for 8 min and cleared by centrifugation at 13,000× *g* at room temperature for 20 min. Equal amounts of proteins (15 µg) were loaded on 10% SDS PAGE gels. Immunoblotting analysis and chemiluminiscence signal development were carried out according to [77]. As primary antibodies, anti-CSD2, anti-PrxQ, anti-ACTIN 2, 8 and 11; all from Agrisera (Vännäs, Sweden) were used diluted 1:3000, 1:1000 and 1:5000, respectively, in Tris-buffered saline containing 0.1% (*v*/*v*) Tween-20. The band optical densities were quantified using Image J. Analyses were performed in three biological replicates.

### 4.8. TBARS Assay

The plant material for TBARS assay was prepared as described in Section 4.6. In this analysis, lipid peroxidation was assayed using the TBARS (thiobarbituric acid reactive substances) assay as described in [78].

### 4.9. Modelling of ACTIN2 Protein Structure

Samples of genomic DNA from the *der1–3* mutant plants (from three different samples) were isolated using a phenol/chloroform/isoamylalcohol protocol [79]. Isolated genomic DNA samples were subjected to sequencing (SeqMe, Dobříš, Czech Republic). Acquired sequences were compared with control *ACTIN2* genomic DNA sequence in Nucleotide BLAST database (BLAST, U.S. National Library of Medicine, National Center for Biotechnology Information; https://blast.ncbi.nlm.nih.gov/Blast.cgi?PROGRAM=blastn&PAGE_TYPE=BlastSearch&LINK_LOC=blasthome, accessed on 12 February 2021). Sequences (both control and mutated) were translated to protein sequences in the application available at http://bio.lundberg.gu.se, accessed on 12 February 2021, (University of Gothenburg, Sweden; http://bio.lundberg.gu.se/edu/translat.html?fbclid=IwAR3var5FJ8CBl4QqNe4Yic8NVz0TvWRd0TrFuGUo6Nk6idLQxy2HvQqPEU, accessed on 12 February 2021). Only one single point mutation found (1114 C-T) changed the protein sequence (Arg97-Cys97) accordingly. Protein sequences were used for protein structure modelling using the application SWISS-MODEL (Biozentrum, University of Basel, Switzerland; https://swissmodel.expasy.org/interactive?fbclid=IwAR1V9lhUgjiR1kUwFLd8ojFftkHpkZwxIoT6mnEVulEC2cPSYQov2twoE, accessed on 12 February 2021). The same application was used also for the generation and downloading of representative images and videos.

### 4.10. Data Acquisition and Analysis

Evaluated parameters such as root growth, skewness (representing an extent of actin filament bundling) and actin filament fluorescence integrated density (representing a percentage of occupancy) were measured in ImageJ (http://rsb.info.nih.gov/ij/, accessed on 12 February 2021). Graphs were prepared in the Microsoft Excel program. Statistical significance between treatments at *p* < 0.05 was performed using a *t*-Test in Microsoft Excel or in program the STATISTICA 12 (StatSoft, TIBCO Software Inc., Palo Alto, CA, USA) by ANOVA and subsequent Fisher’s LSD test (*p* < 0.05).

## Data Availability

The data presented in this study are available in the article and supplementary material.

## Supplementary Materials

The following materials are available online at https://www.mdpi.com/1422-0067/22/4/1879/s1, Figure S1: Impact of PQ treatment on the root growth rate in control and transgenic C24 and *der1–3* mutant lines, Figure S2: Effect of PQ treatment on the distance between the first root hair and the root tip in control and transgenic C24 and *der1–3* mutant lines, Figure S3: Root growth rate in plants of control C24, *der1–3* mutant and transgenic C24 and *der1–3* lines under prolonged PQ treatment, Figure S4: Shoot and root fresh weight in plants of control C24, *der1–3* mutant and transgenic C24 and *der1–3* lines expressing *pro35S::GFP:FABD2* after germination and growth in PQ-containing media, Figure S5: Root growth rate in control and transgenic C24 and *der1–3* mutant lines after their transfer to PQ-containing media, Figure S6: Plant phenotype of control and transgenic C24 and *der1–3* mutant lines on the control media, Figure S7: Phenotype of control C24 and *der1–3* mutant plants after their transfer to H_2_O_2_-containing media, Figure S8: Histochemical detection of O_2_^•−^ and H_2_O_2_ production in the cotyledons and leaves of control C24 and *der1–3* mutant plants after their transfer to PQ- and H_2_O_2_-containing media, Figure S9: Full scan of the entire original gel stained for specific activity of superoxide dismutases presented in Figure 7B, Figure S10: Full scan of the entire original immunoblots presented in Figure 7, Figure S11: Full scans of the entire original immunoblots presented in Figure 8, Video S1: 3D rotational model of protein structure of the nature ACTIN2 protein, Video S2: 3D rotational model of protein structure of mutated version of ACTIN2 in *der1–3* mutant, Video S3: Actin filaments in hypocotyl epidermal cells of C24 GFP-FABD2 line recorded in 30 s intervals for 30 min in control conditions, Video S4: Actin filaments in hypocotyl epidermal cells of C24 GFP-FABD2 line recorded in 30 s intervals after treatment with 0.1 µmol·L^−1^ of PQ for 30 min, Video S5: Actin filaments in hypocotyl epidermal cells of the *der1–3* GFP-FABD2 line recorded in 30 s intervals for 30 min in control conditions, Video S6: Actin filaments in hypocotyl epidermal cells of *der1–3* GFP-FABD2 line recorded in 30 s intervals after treatment with 0.1 µmol·L^−1^ of PQ for 30 min.
