# Supplementary material for: Single Amino Acid Exchange in ACTIN2 Confers Increased Tolerance to Oxidative Stress in Arabidopsis der1–3 Mutant
Source: Int J Mol Sci. 2021 Feb 13;22(4):1879. doi: 10.3390/ijms22041879 (PMC7918201; doi:10.3390/ijms22041879)
Supplement: Supplementary file 1 [file ijms-22-01879-s001.zip › Kubenova et al. Supplementary files/Kubenova et al. Supplementary Figures.pdf]

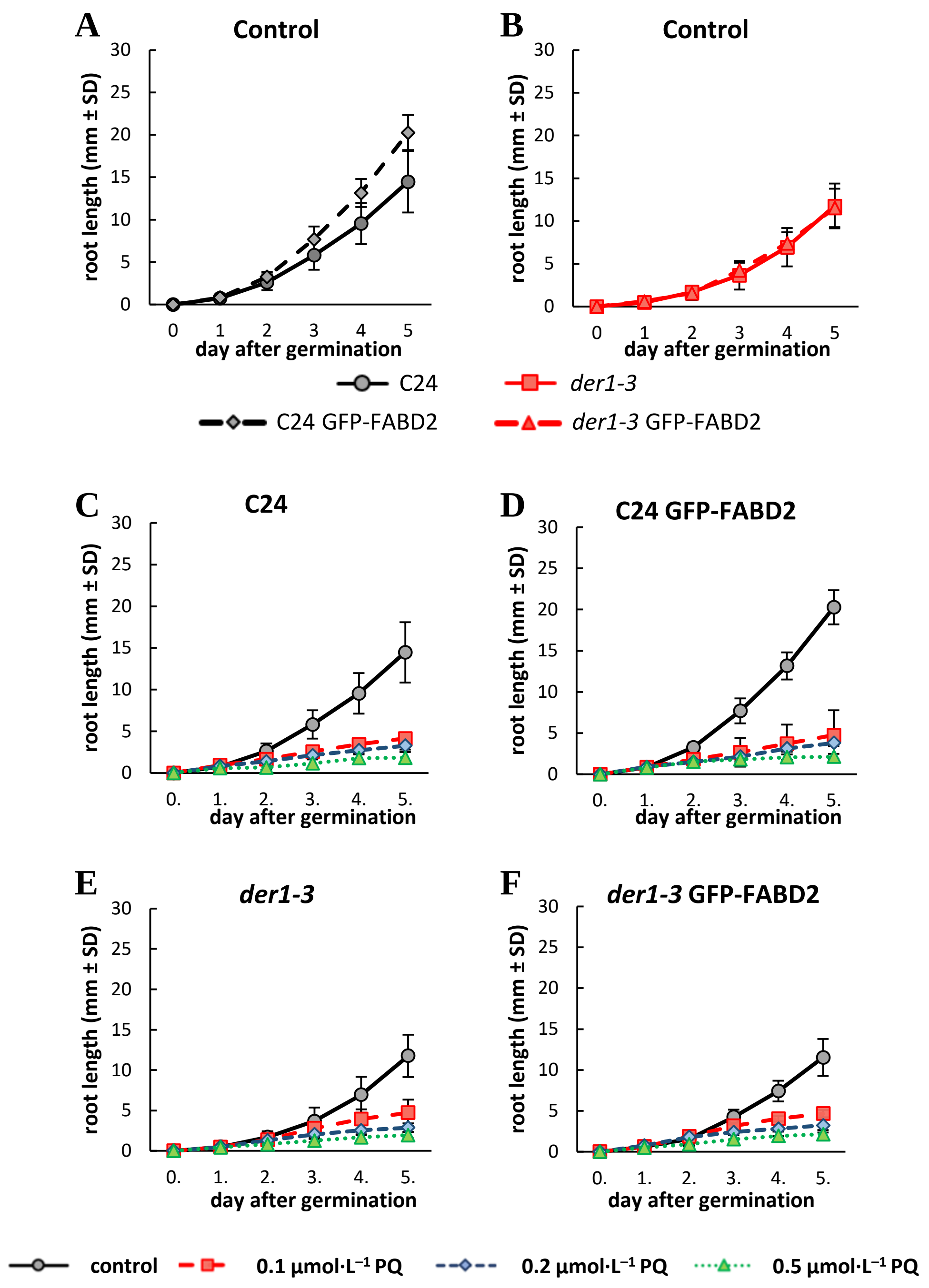


**Figure S1. Impact of PQ treatment on root growth rate in control and transgenic C24 and *der1-3* mutant lines.** **(A-B)** Comparison of root growth rate within the first 5 days after germination on control media between control C24 and transgenic C24 line carrying GFP-FABD2 **(A)** and between *der1-3* and transgenic *der1-3* line carrying GFP-FABD2 **(B)**. **(C-F)** Root growth rate of control C24 **(C)**, *der1-3* mutant **(E)**, as well as transgenic C24 **(D)** and transgenic *der1-3* **(F)** lines carrying GFP-FABD2 within the first 5 days after germination on control media and media containing 0.1, 0.2 and 0.5 µmol·$L^{-1}$ PQ. Experiments were repeated twice with 16 seeds per line in control conditions and with 12 seeds per line under PQ treatment.

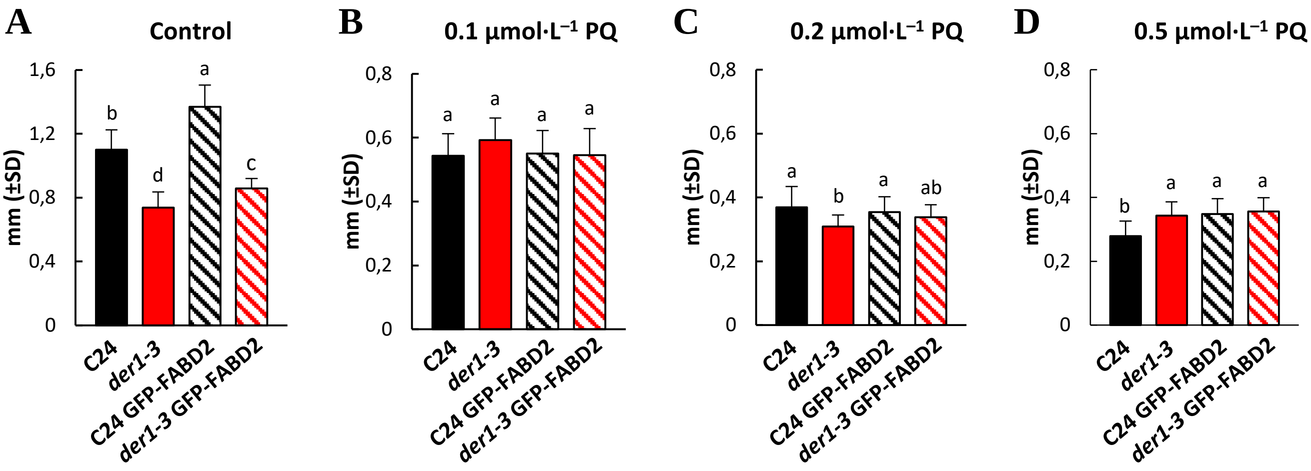


**Figure S2. Effect of PQ treatment on the distance between the first root hair and the root tip in control and transgenic C24 and *der1-3* mutant lines.** The distance of the first root hair from the root tip was analysed in roots of 5 days-old plants of control C24, *der1-3* mutant, transgenic C24 line carrying GFP-FABD2 and transgenic *der1-3* line carrying GFP-FABD2 germinated and grown on control media **(A)** and media containing 0.1 **(B)**, 0.2 **(C)** and 0.5 **(D)** $\mu mol \cdot L^{-1}$ PQ. Experiments were repeated twice with 16 seeds per line in control conditions and with 12 seeds per line under PQ treatment. Different lowercase letters above the bars represent statistical significance according to one-way ANOVA and subsequent LSD test at $p$ value $< 0.05$.

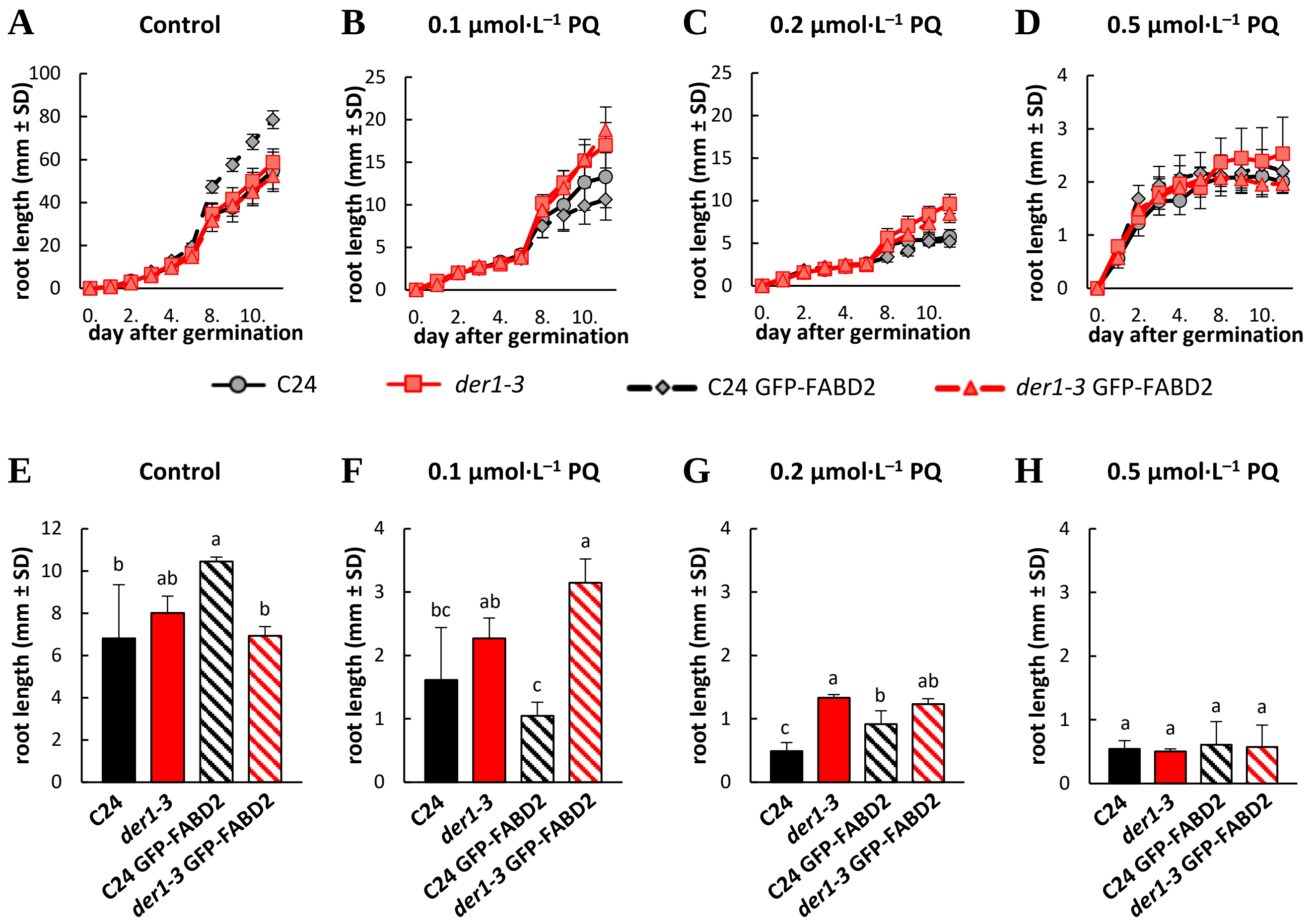


**Figure S3. Root growth rate in plants of control C24, *der1-3* mutant and transgenic C24 and *der1-3* lines under prolonged treatment with PQ. (A-D)** Growth rate of the main root in plants of control C24, *der1-3* mutant, transgenic C24 line carrying GFP-FABD2 and transgenic *der1-3* line carrying GFP-FABD2 on control media **(A)** and on media containing 0.1 **(B)**, 0.2 **(C)** and 0.5 **(D)** $\mu mol \cdot L^{-1}$ PQ within 11 days after germination. **(E-H)** Average root growth per 24h on the control media **(E)** and on media containing 0.1 **(F)**, 0.2 **(G)** and 0.5 **(H)** $\mu mol \cdot L^{-1}$ PQ. Experiments were done with 15 plants per line. Different lowercase letters above the bars **(E-H)** represent statistical significance according to one-way ANOVA and subsequent LSD test at $p$ value $< 0.05$.

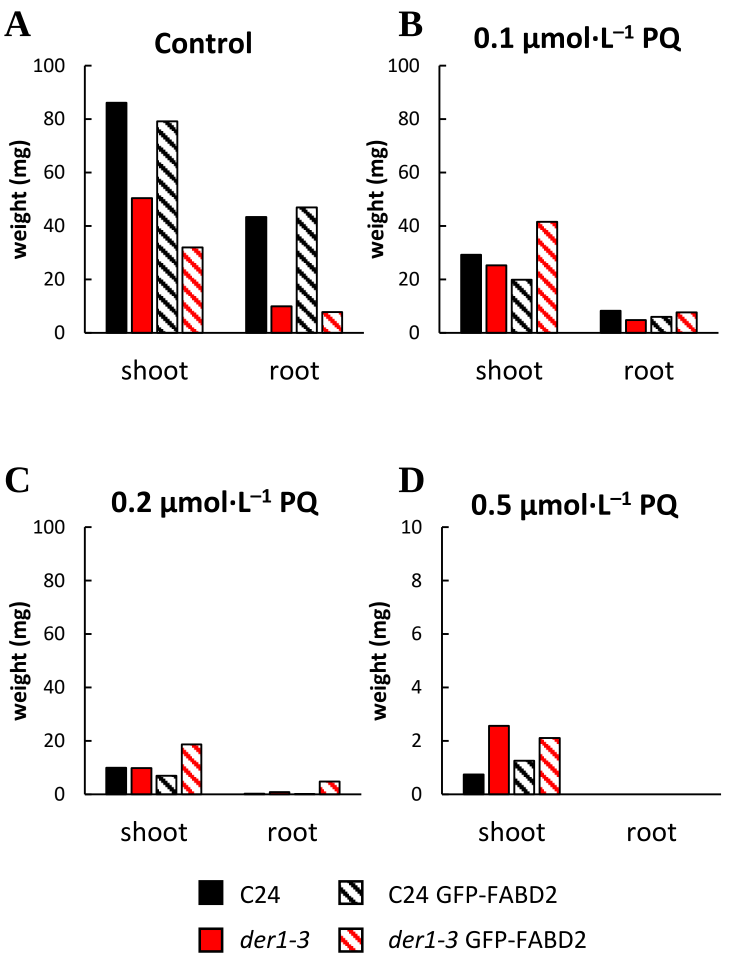


**E**

| 0.1 µmol·L$^{-1}$ PQ | shoot | root |
|---|---|---|
| C24 | 2.94 | 5.22 |
| *der1-3* | 2.00 | 2.08 |
| C24 GFP-FABD2 | 3.98 | 7.75 |
| *der1-3* GFP-FABD2 | 0.77 | 1.01 |

**F**

| 0.2 µmol·L$^{-1}$ PQ | shoot | root |
|---|---|---|
| C24 | 8.67 | 139.97 |
| *der1-3* | 5.15 | 11.86 |
| C24 GFP-FABD2 | 11.35 | 261.22 |
| *der1-3* GFP-FABD2 | 1.71 | 1.62 |

**G**

| 0.5 µmol·L$^{-1}$ PQ | shoot |
|---|---|
| C24 | 114.96 |
| *der1-3* | 19.71 |
| C24 GFP-FABD2 | 62.87 |
| *der1-3* GFP-FABD2 | 15.17 |

**Figure S4. Shoot and root fresh weight in plants of control C24, *der1-3* mutant and transgenic C24 and *der1-3* lines expressing *pro35S::GFP:FABD2* after germination and growth in PQ-containing media. (A-D)** Fresh weight (pooled shoots and pooled roots, respectively) from plants of control C24, *der1-3* mutant, transgenic C24 line carrying GFP-FABD2 and transgenic *der1-3* line carrying GFP-FABD2 growing 18 days on control media **(A)** and on media containing 0.1 **(B)**, 0.2 **(C)** and 0.5 **(D)** µmol·L$^{-1}$ PQ. **(E-G)** Reduction ratio (fold change in respect to control) of shoot and root fresh weight of respective lines growing on media containing 0.1 **(E)**, 0.2 **(F)** and 0.5 **(G)** µmol·L$^{-1}$ PQ. Experiments were repeated two times with 16 plants per line (control) and 12 plants per line (PQ).

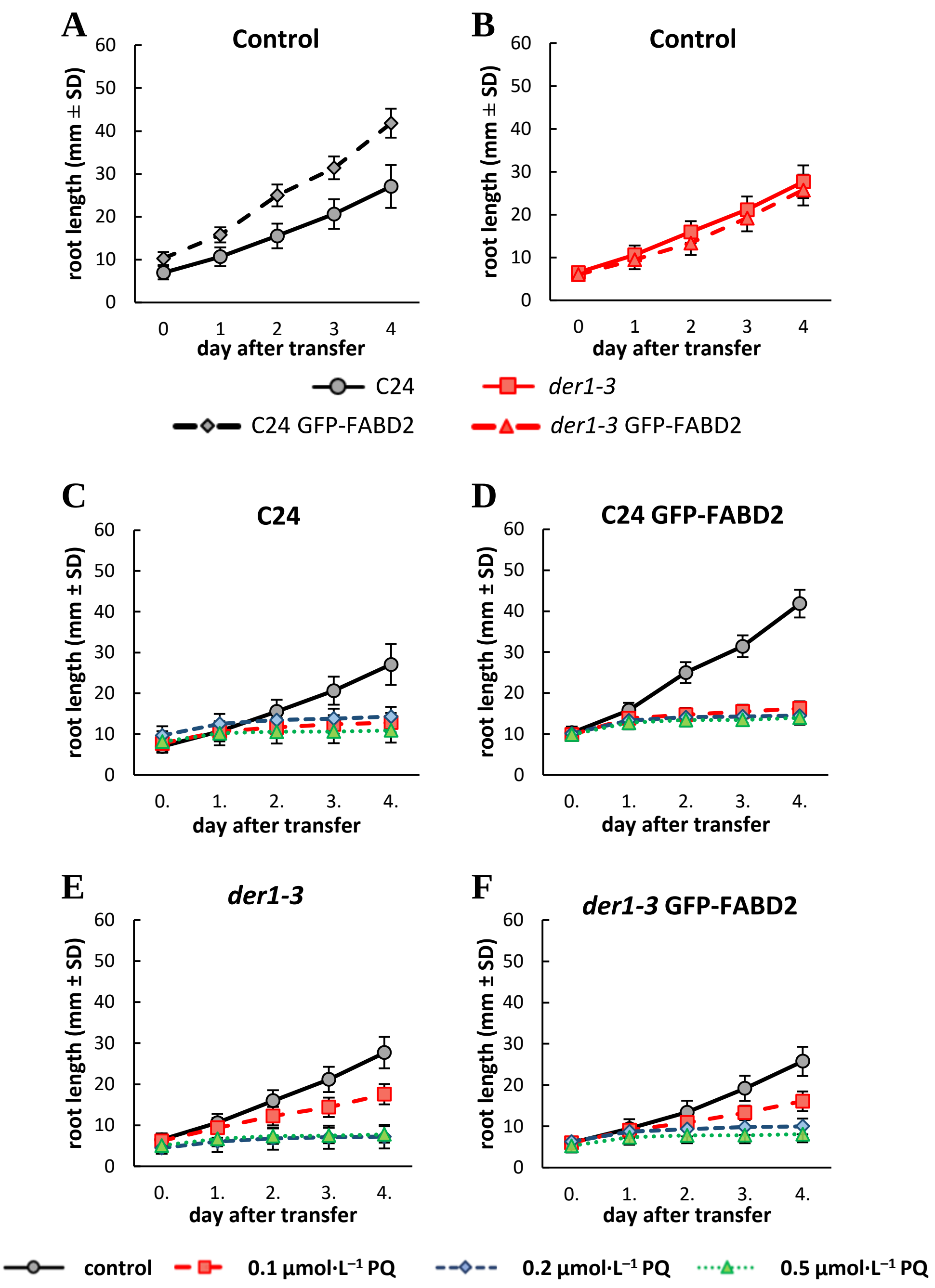


**Figure S5. Root growth rate in control and transgenic C24 and *der1-3* mutant lines after their transfer to PQ-containing media. (A-B)** Comparison of root growth rate within the first 4 days after transfer to control media between control C24 and transgenic C24 line carrying GFP-FABD2 **(A)** and between *der1-3* and transgenic *der1-3* line carrying GFP-FABD2 **(B)**. **(C-F)** Root growth rate of control C24 **(C)**, *der1-3* mutant **(E)**, as well as transgenic C24 **(D)** and transgenic *der1-3* **(F)** lines carrying GFP-FABD2 within the first 4 days after transfer to control media and media containing 0.1, 0.2 and 0.5 µmol·L$^{-1}$ PQ. Experiments were repeated twice with 16 seeds per line in control conditions and with 12 seeds per line under PQ treatment.

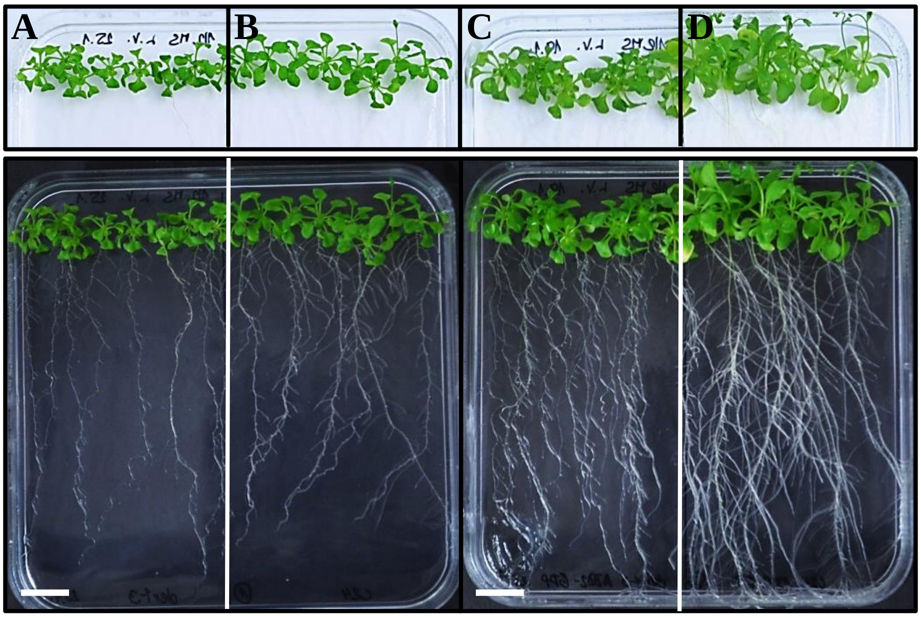


**Figure S6. Plant phenotype of control and transgenic C24 and *der1-3* mutant lines on control media.** Plants of *der1-3* mutant **(A)**, control C24 **(B)**, transgenic *der1-3* line carrying GFP-FABD2 **(C)** and transgenic C24 line carrying GFP-FABD2 **(D)**. Aboveground parts of plants were photographed on white background (upper row of images), and whole plants including roots were documented on black background (lower row of images). Plants germinated and grown on control media for 20 days. Scale bar = 1 cm.

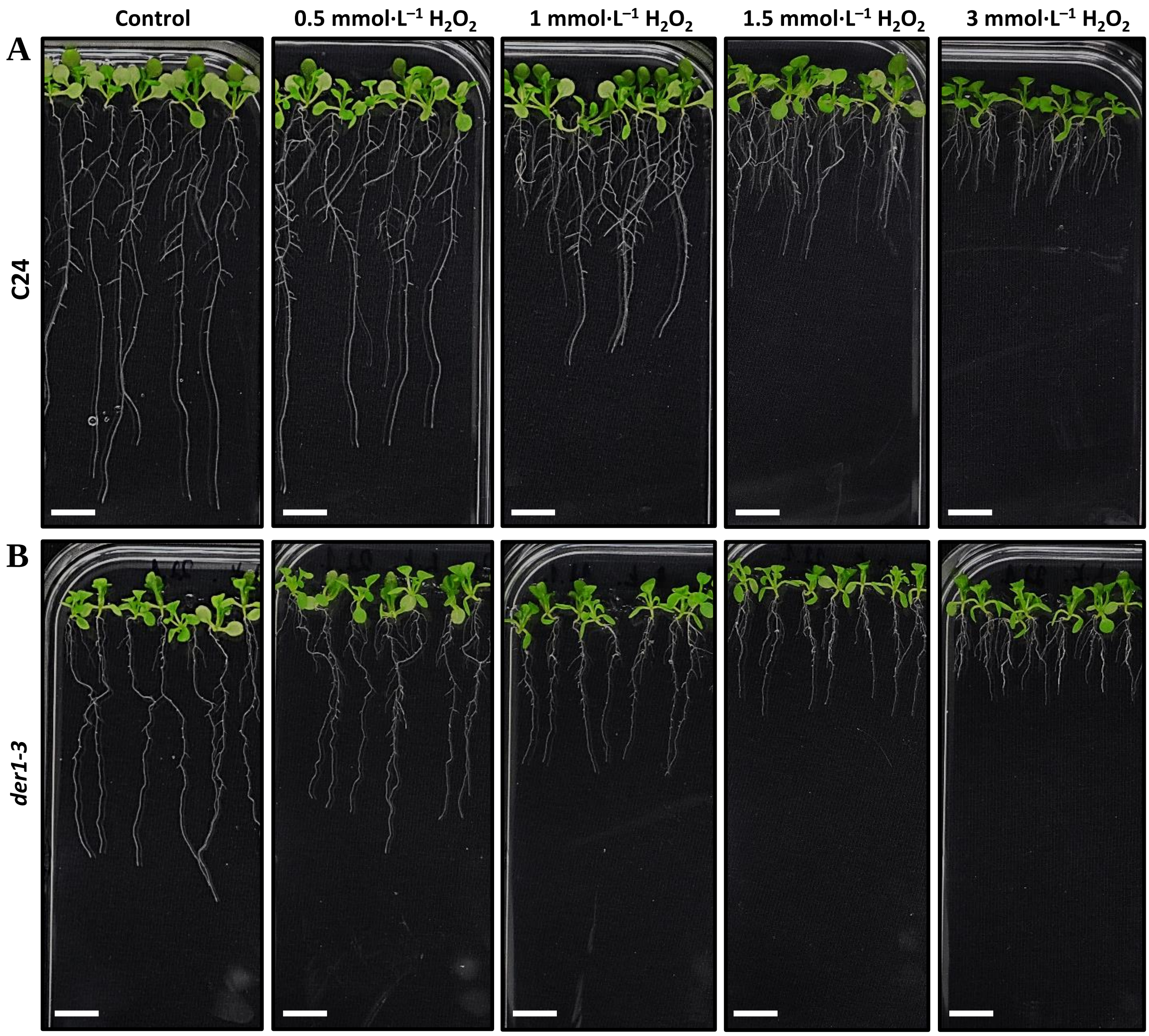


**Figure S7. Phenotype of control C24 and *der1-3* mutant plants after their transfer to $H_2O_2$-containing media.** Plants 3 days old germinated on control media were transferred to $H_2O_2$-containing media and photographed 8 days after transfer. **(A-B)** Plants of control C24 **(A)** and *der1-3* mutant **(B)** growing on media containing 0.5, 1, 1.5 and 3 mmol·$L^{-1}$ $H_2O_2$. Scale bar = 1 cm.

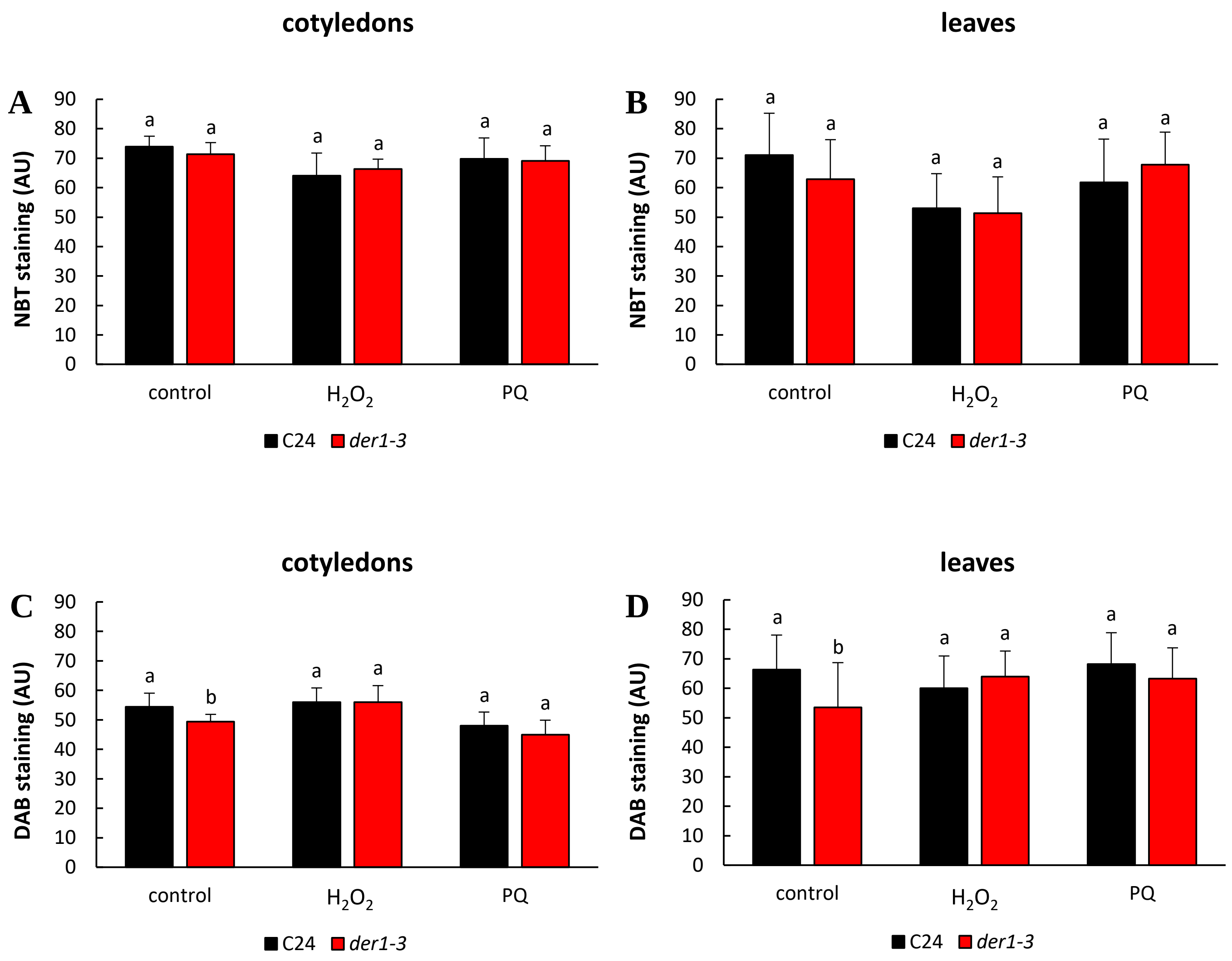


**Figure S8. Histochemical detection of $O_2^{\bullet-}$ and $H_2O_2$ production in cotyledons and leaves of control C24 and *der1-3* mutant plants after their transfer to PQ- and $H_2O_2$-containing media.** Plants 3 days old germinated on control media were transferred to PQ- and $H_2O_2$-containing media and analysed 11 days after transfer. **(A-B)** Nitroblue tetrazolium (NBT) staining in cotyledons **(A)** and leaves **(B)**. **(C-D)** 3,3′-diaminobenzidine (DAB) staining in cotyledons **(C)** and leaves **(D)**. Results are presented as mean intensity of the histochemical staining from plants transferred to media containing 0.1 µmol.$L^{-1}$ PQ and 3 mmol·$L^{-1}$ $H_2O_2$.

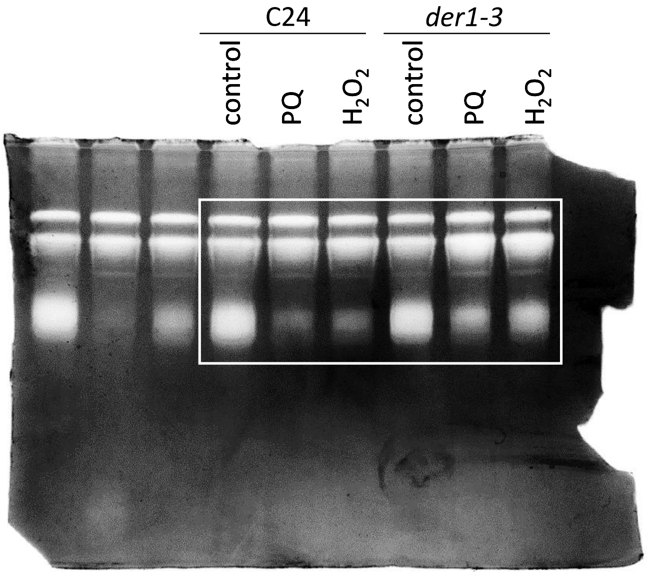


**Figure S9.** Full scan of the entire original gel stained for specific activity of superoxide dismutases presented in Figure 7B. The highlighted region shows the presented section. Samples loaded on lanes which are not annotated are not relevant to this study.

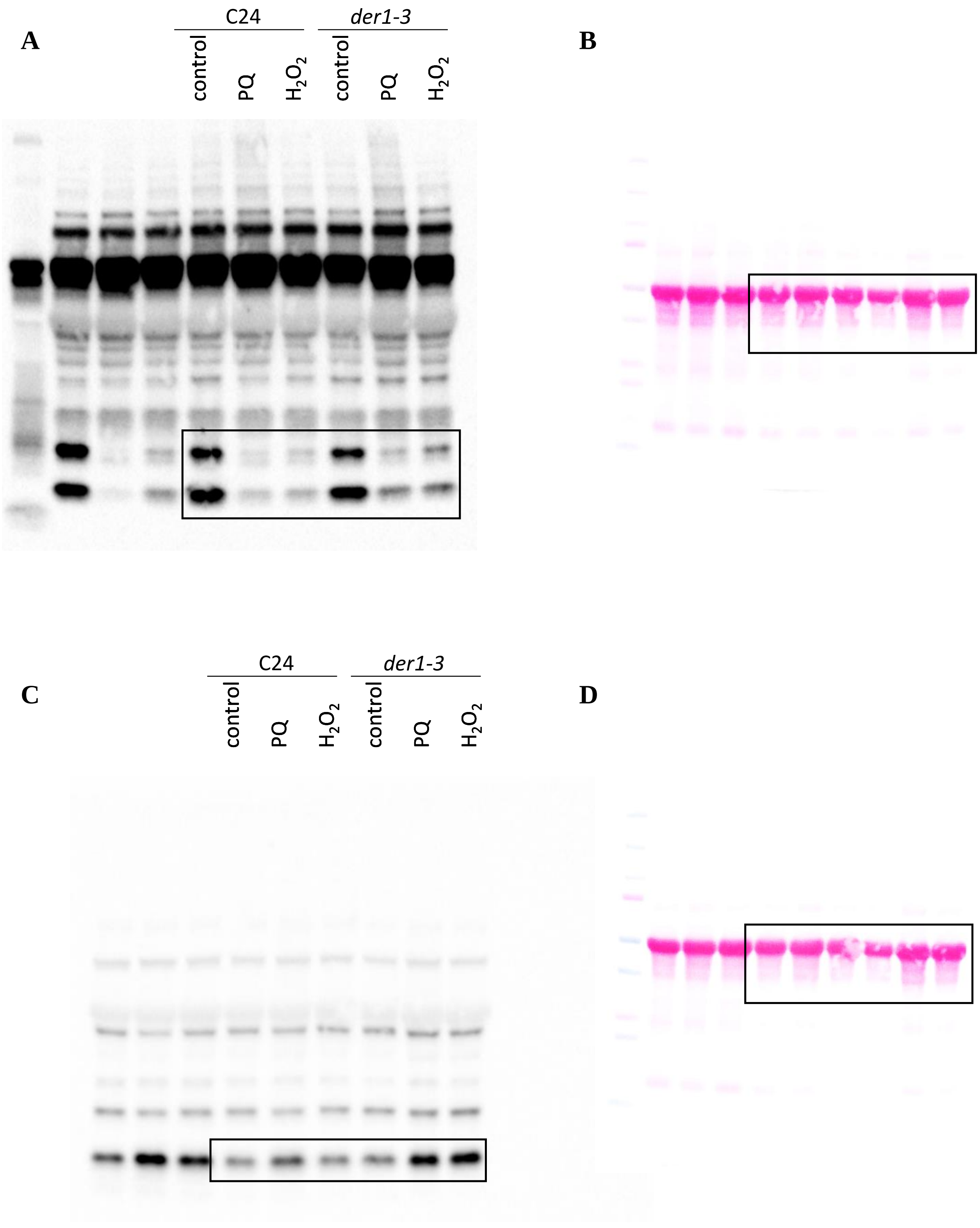


**Figure S10.** Full scan of the entire original immunoblots presented in Figure 7. **(A)** Entire membrane with chemiluminiscent signal observed after probing with anti-CSD2 antibody. The highlighted region shows the section presented in Figure 7D. **(B)** Full image of the membrane in **(A)**, after staining with Ponceau S. The highlighted region shows the section presented in Figure 7D. **(C)** Entire membrane with chemiluminiscent signal observed after probing with anti-PrxQ antibody. The highlighted region shows the section presented in Figure 7F. **(D)** Full image of the membrane in **(C)**, after staining with Ponceau S. The highlighted region shows the section presented in Figure 7F. Samples loaded on lanes which are not annotated are not relevant to this study.

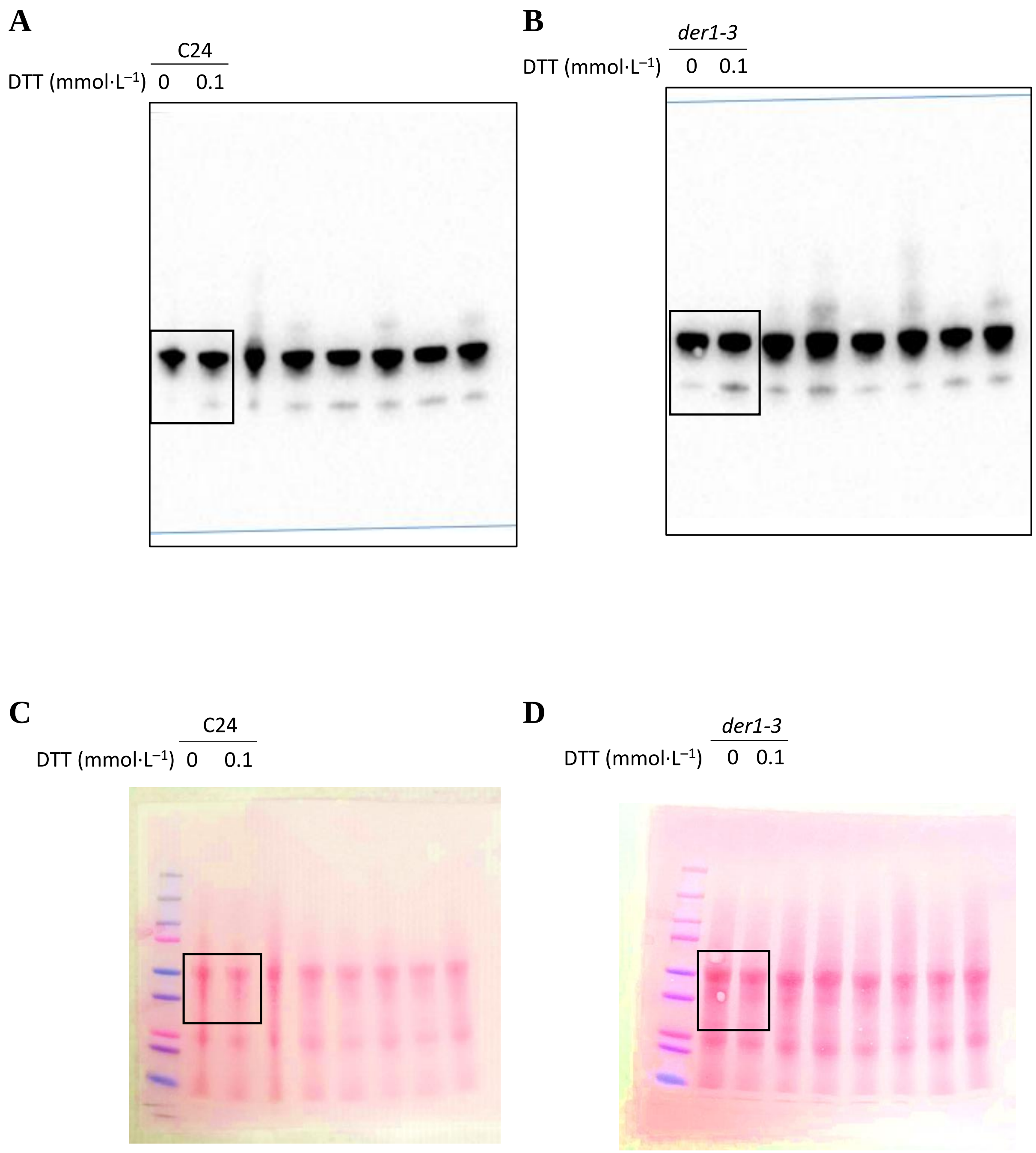


**Figure S11**. Full scans of the entire original immunoblots presented in Figure 8. **(A-B)** Entire membranes with chemiluminiscent signal observed after probing with anti-ACTIN 2,8,11 antibody. The highlighted regions show the sections presented in Figures 8A, B. **(C-D)** Full images of the membranes in **(A, B)**, after staining with Ponceau S. The highlighted regions show sections presented in Figures 8A, B. Note that lanes which are not annotated are not relevant to present study.
